# Novel personalized cancer vaccine platform based on Bacillus Calmette-Guèrin

**DOI:** 10.1136/jitc-2021-002707

**Published:** 2021-07-15

**Authors:** Erkko Ylösmäki, Manlio Fusciello, Beatriz Martins, Sara Feola, Firas Hamdan, Jacopo Chiaro, Leena Ylösmäki, Matthew J Vaughan, Tapani Viitala, Prasad S Kulkarni, Vincenzo Cerullo

**Affiliations:** 1 Laboratory of Immunovirotherapy, Drug Research Program, Faculty of Pharmacy, University of Helsinki, Helsinki, Finland; 2 TRIMM, Translational Immunology Research Program, University of Helsinki, Helsinki, Finland; 3 Valo Therapeutics Oy, Helsinki, Finland; 4 Pharmaceutical Biophysics Research Group, Drug Research Program, Faculty of Pharmacy, University of Helsinki, Helsinki, Finland; 5 Serum Institute of India Pvt Ltd, Pune, India; 6 iCAN Digital Precision Cancer Medicine Flagship, University of Helsinki, Helsinki, Finland; 7 Department of Molecular Medicine and Medical Biotechnology and CEINGE, Naples University 24 Federico II, Naples, Italy

**Keywords:** adaptive immunity, immunity, cellular, immunogenicity, vaccine, immunotherapy, immunotherapy, active

## Abstract

**Background:**

Intratumoral BCG therapy, one of the earliest immunotherapies, can lead to infiltration of immune cells into a treated tumor. However, an increase in the number of BCG-induced tumor-specific T cells in the tumor microenvironment could lead to enhanced therapeutic effects.

**Methods:**

Here, we have developed a novel cancer vaccine platform based on BCG that can broaden BCG-induced immune responses to include tumor antigens. By physically attaching tumor-specific peptides onto the mycobacterial outer membrane, we were able to induce strong systemic and intratumoral T cell-specific immune responses toward the attached tumor antigens. These therapeutic peptides can be efficiently attached to the mycobacterial outer membrane using a poly-lysine sequence N-terminally fused to the tumor-specific peptides.

**Results:**

Using two mouse models of melanoma and a mouse model of colorectal cancer, we observed that the antitumor immune responses of BCG could be improved by coating the BCG with tumor-specific peptides. In addition, by combining this novel cancer vaccine platform with anti-programmed death 1 (anti-PD-1) immune checkpoint inhibitor (ICI) therapy, the number of responders to anti-PD-1 immunotherapy was markedly increased.

**Conclusions:**

This study shows that intratumoral BCG immunotherapy can be improved by coating the bacteria with modified tumor-specific peptides. In addition, this improved BCG immunotherapy can be combined with ICI therapy to obtain enhanced tumor growth control. These results warrant clinical testing of this novel cancer vaccine platform.

## Introduction

BCG, a live attenuated strain of *Mycobacterium bovis*, is currently the treatment of choice for urothelial carcinoma in situ (CIS) of the bladder.^1 2^ BCG has also been used previously as an intralesional monotherapy for in-transit melanoma that has resulted, in some studies, in up to 90% regression of BCG-injected lesions and 17% regression of uninjected lesions in immunocompetent patients.^3–5^ In addition, intralesional treatment with BCG has been combined with topical imiquimod (a toll-like receptor (TLR) 7 agonist) treatment resulting in a complete response rate of 56%.^6 7^ BCG is an intracellular pathogen that can modulate the tumor microenvironment (TME) by multiple mechanisms including an induction of a massive secretion of chemokines and cytokines that recruit T cells and other immune cells to the TME, as well as by polarization of M2 macrophages toward a more M1-like phenotype.^8 9^ Recently, it was shown that BCG treatment led to enhanced activation and reduced exhaustion of tumor-specific T cells, leading to enhanced effector functions and that BCG-induced bladder cancer elimination required tumor-specific CD4^+^ and CD8^+^ T cells, but not T cells specific for BCG antigens.^10^

Another class of cancer immunotherapy, immune checkpoint inhibitors (ICIs), using antibodies targeting immune checkpoint molecules such as programmed death 1 (PD-1), programmed death-ligand 1 (PD-L1) and cytotoxic T-lymphocyte-associated protein 4 (CTLA-4) have demonstrated induction of long-term tumor regression and durable responses in some patients with cancer, with response rates of 10%–25% in the majority of cancers.^11^ Patients responding to ICI therapy seem to have a pre-existing antitumor immune response with immune cell infiltration into the tumor, which is then enhanced and rendered functional by ICI therapy.^12 13^ As a consequence, novel combinational therapies that attract tumor-specific CD8^+^ T cells into tumors to increase the number of responders to ICI therapy are much needed.

In order to increase BCG-induced tumor-specific T cell responses and the antitumor effects of BCG therapy when combined with ICI therapy, we developed a cancer vaccine platform based on coating BCG bacteria with tumor-specific peptides for broadening the immune response to include the treated tumor as well. Intratumoral administration of this cancer vaccine platform, named PeptiBAC (**pepti**de-coated **Bac**illus Calmette-Guérin) as a monotherapy, increased systemic tumor-specific T cell responses in two mouse models melanoma. When used in combination with an ICI against PD-1, PeptiBAC reduced tumor growth, increased tumor-specific intratumoral as well as systemic T cell responses, and sensitized tumors to anti-PD-1 ICI therapy by increasing the number of mice responsive to the combination treatment (PeptiBAC in combination with anti-PD-1 ICI). The PeptiBAC platform was also tested in combination with our recently described cancer vaccine platform PeptiCRAd^14^ (**pepti**de-coated **c**onditionally **r**eplicating **ad**enovirus) using a heterologous prime-boost vaccination strategy.^15^ The heterologous PeptiBAC prime–PeptiCRAd boost vaccination increased tumor-specific T cell immune responses by directing the immune responses toward the tumor-specific peptides. The elegance of this platform is the introduction of antitumor immunity-inducing peptides non-genetically to the BCG vaccine, which makes this approach highly adaptable and thus suitable for personalized immunotherapeutic approaches that rely on the identification of patient-specific neo-antigens.

## Materials and methods

### Cell lines and reagents

Murine colon carcinoma CT26.wt cell line was purchased from American Type Culture Collection (ATCC) and was cultured in high glucose RPMI with 10% fetal calf serum (FBS) (Life Technologies), 1% L-glutamine and 1% penicillin/streptomycin. B16F10.9/K1 cell line was kindly provided by Ludovic Martinet (Inserm, France) and was cultured in high glucose Dulbecco’s Modified Eagle Medium (DMEM) supplemented with 10% FBS, 1% L-glutamine and 1% penicillin/streptomycin. The cell line B16.OVA, a mouse melanoma cell line expressing chicken ovalbumin (OVA), was kindly provided by Professor Richard Vile (Mayo Clinic, Rochester, Minnesota, USA). B16.OVA cells were cultured in DMEM with 10% FBS (Life Technologies), 1% L-glutamine, 1% penicillin/streptomycin and 5 mg/mL of geneticin. Murine dendritic cell (DC) line JAWSII was purchased from ATCC and was cultured in alpha minimum essential medium with 20% FBS (Life Technologies), ribonucleosides, deoxyribonucleosides, 4 mM L-glutamine (Life Technologies), 1 mM sodium pyruvate (Life Technologies), and 5 ng/mL murine GM-CSF (PeproTech, USA). Murine macrophage reporter cell line RAW-Blue (InvivoGen) was cultured in DMEM supplemented with 10% FBS, 1% L-glutamine, 1% penicillin/streptomycin, 100 µg/mL Normocin (InvivoGen) and 100 µg/mL Zeocin (InvivoGen) as a selective antibiotic. Human lung carcinoma A549 cell line was purchased from National Institutes of Health (NIH) and was cultured in OptiPRO SFM supplemented with 10% FBS (Life Technologies), 1% L-glutamine and 1% penicillin/streptomycin. All cells were cultured at 37 °C/5% CO_2_ and were routinely tested for mycoplasma contamination using a commercial detection kit (Lonza).

### Bacteria

Live attenuated BCG vaccines were obtained from various sources. SII BCG (2–8×10^6^ colony forming units (CFU)/vial) and SII-ONCO-BCG vaccine (1–19.2×10^8^ CFU/vial) were kindly provided by the Serum Institute of India (Pune, India). BCG vaccine (1.5–6.0×10^6^ CFU/vial) was purchased from InterVax (Toronto, Canada), while BCG vaccine AJV (2–8×10^6^ CFU/vial) from AJ Vaccines (Copenhagen, Denmark) was a kind gift from Professor Helen McShane (University of Oxford).

### Viruses

An adenovirus expressing murine OX40L and CD40L (VALO-mD901) was used in heterologous prime-boost experiments. The development of VALO-mD901 has previously been described.^16^ Briefly, a part of the E3B region of a pAd5/3-D24 backbone plasmid was replaced with human cytomegalovirus (CMV) promoter region, murine OX40L, a 2A self-cleaving peptide sequence, murine CD40L gene and rabbit β-globin polyadenylation signal. The virus was amplified in A549 cells and purified on double cesium chloride gradients and stored below −60°C in A195 adenoviral storage buffer.^17^ The viral particle (VP) concentration was measured at 260/280 nm and infectious units (IU) were determined by immunocytochemistry by staining the hexon protein on A549-infected cells.

### Peptides

The following peptides were used in this study: GRKKRRQRRRPQRWEKISIINFEKL, RWEKISIINFEKL, KKKKKK-SIINFEKL and SIINFEKL (containing a major histocompatibility complex (MHC) class I-restricted epitope from chicken ovalbumin, OVA_257-264_), KKKKKK-SVYDFFVWL and SVYDFFVWL (containing an MHC class I-restricted epitope from tyrosinase-related protein 2, Trp2_180–188_), KKKKKK-SPSYAYHQF and SPSYAYHQF (containing a modified MHC class I-restricted epitope from murine leukemia virus envelope glycoprotein 70 (gp70_423–431_) where V5A change was made to the original AH1 epitope for enhanced immunogenicity).^18^ All peptides were purchased from Zhejiang Ontores Biotechnologies (Zhejiang, China).

### PeptiBAC complex formation

0.75×10^5^–12×10^7^ CFU of BCG resuspended in PBS were complexed with 40–90 nmol of CPP or polyK-extended peptides resuspended in dimethyl sulfoxide (DMSO) and incubated for 15 min at room temperature (RT). After complexation, PeptiBAC complexes were pelleted by centrifugation at 1000×g for 10 min at RT and the buffer was changed to remove unbound peptides.

### PeptiCRAd complex formation

PeptiCRAd complexes were prepared by mixing VALO-mD901 adenovirus (in A195 storage buffer) with polyK-extended Trp2 epitope (in 0.9% saline) at a ratio of 1.8×10^5^ peptides per one virus particle. The mixture was then incubated at RT for 15 min. For animal injections, the complexes were diluted further in 0.9% saline to administration volume.

### Surface plasmon resonance

Measurements were performed using a multi-parametric surface plasmon resonance (SPR) Navi 220A instrument (Bionavis, Tampere, Finland). Phosphate buffered saline (PBS) (pH 7.4) was used as a running buffer. A constant flow rate of 20 µL/min was used throughout the experiments, and temperature was set to +20°C. Laser light with a wavelength of 670 nm was used for surface plasmon excitation. An Au-SiO_2_ sensor slide was activated by 5 min of plasma treatment followed by coating with APTES ((3-aminopropyl) triethoxysilane) by incubating the sensor in 50 mM APTES in isopropanol for 4 hours. The sensor was then washed and placed into the SPR device. BCG was immobilized in situ on the sensor surface in two of four test channels by injecting approximately 1–4×10^6^ CFU of BCG in PBS (pH 7.4) for 12 min, followed by a 3 min wash with PBS. For testing the interaction between various peptides and the mycobacterial outer membrane, 100 µM of the tested peptides extended with CPP or poly-lysine sequences, or without the attachment moieties (as non-interacting controls) was injected into a BCG-coated channel and into an uncoated channel of the flow cell.

The number of peptides per BCG particle was estimated according to the following procedure:

1. First, it was assumed that a fully covered sensor surface forms a monolayer of hexagonally packed layer of BCG particles. This means that only 74% of the sensor surface can be covered by the bacteria (based on geometrical calculations). For this, the average length (2.36 µm) and width (0.47 µm) of a BCG bacterium was converted to a spherical particle with a volume of 0.3887 μm^3^ and a diameter of 905.5 nm.
2. In order to estimate the thickness of a hexagonally packed layer of BCG particles, we performed optical modeling of the SPR sensor properties for a plain sensor without BCG and a sensor fully covered with a layer of BCG particles. However, in optical modeling of the SPR sensor properties, we needed to consider that the models assume even homogeneous layers without spaces and thus we converted the volume of a sphere to the corresponding value of a cube by using a conversion factor of 0.524 (based on geometrical calculations).
3. In order to estimate the theoretical even homogeneous thickness of a fully covered hexagonally packed BCG layer, we first multiplied the average diameter of BCG with 0.74 (contribution from hexagonal packing) and then with 0.524 (contribution of filling the gaps between spheres into a homogeneous even layer).
4. In this way, we obtained a theoretical even homogeneous thickness of a fully covered hexagonally packed layer for BCG of 351.1 nm (assuming an average diameter of 905.5 nm).
5. Hereafter, we calculated through optical modeling the maximum SPR angular response induced by this BCG layer by assuming a refractive index of 1.35 for BCG and obtained 2.28° (see online supplemental figure 6).
6. The actual measured SPR response during immobilization of the BCG on the SPR sensor surface was then divided with the corresponding maximum SPR angular response modeled for a monolayer of hexagonally packed layer of BCG (ie, 2.28°). This ratio was then assumed to reflect the percentage of the detection area covered with BCG. For the measurements for the different peptides used in this study, the corresponding percentages were 22.6% (6K-AH1 peptide), 13.6% (6K-TRP2) and 11.0% (CPP-SIINFEKL).
7. As the detection area is determined by the diameter of the laser used in the SPR instrument, that is, 1 mm, we were able to calculate the area covered with BCG by multiplying the detection area with the percentage of the detection area covered with BCG (22.6% for the 6K-AH1 peptide, 13.6% for the 6K-TRP2 peptide and 11.0% for the CPP-SIINFEKL peptide).
8. Next, we calculated the footprint area of BCG based on its assumed diameter of 905.5 nm and obtained an area of approximately 643,971 nm^2^ per BCG particle.
9. By dividing the area covering the sensor area with BCG (obtained from point 7) with the footprint area of BCG (obtained from point 8), we obtained the number of BCG particles on the sensor surface. For the measurements for the different peptides in this study, the corresponding number of BCG particles were 275,269 BCG particles (6K-AH1 peptide), 165,397 BCG particles (6K-TRP2 peptide) and 133,729 BCG particles (CPP-SIINFEKL peptide).
10. Hereafter, we calculated the number of peptides adsorbed to BCG from the SPR responses measured when 100 µM of the peptides was allowed to interact with the BCG layers. The SPR response values for the peptides could be converted to mass per area of adsorbed peptides by using a conversion factor of 600 ng/cm^2^×SPR response in degrees. The mass/area determined for the different peptides in this study were: 35.3 ng/cm^2^ (6K-AH1 peptide), 301.6 ng/cm^2^ (6K-TRP2 peptide) and 163.0 ng/cm^2^ (CPP-SIINFEKL peptide).
11. By knowing the detection area, we could estimate the absolute mass of peptides adsorbed on BCG by multiplying the detection area with the mass/area of each peptide. The mass determined for the different peptides in this study were approximately 0.277 ng (6K-AH1 peptide), 2.369 ng (6K-TRP2 peptide) and 1.280 ng (CPP-SIINFEKL peptide).
12. By knowing the molecular weight of the peptides (1868.21 g/mol for 6K-AH1 peptide, 1944.4 g/mol for 6K-TRP2 peptide, 3279.9 g/mol for CPP-SIINFEKL peptide) we were able to convert the mass to moles and finally to number of peptides by using the Avogadro constant. The number of peptides adsorbed for the different peptides in this study are ca. 8.9×10^10^ (6K-AH1 peptide), 7.3×10^11^ (6K-TRP2 peptide) and 2.4×10^11^ (CPP-SIINFEKL peptide).
13. Finally, the number of peptides adsorbed per BCG particle was estimated by dividing the number of peptides (obtained from point 12) with the number of BCG particles obtained (from point 9).

10.1136/jitc-2021-002707.supp1 Supplementary data

10.1136/jitc-2021-002707.supp8 Supplementary data

### DC cross-presentation experiments

JAWSII cells were seeded in 24 well plates (5×10^5^ cells/well) and pulsed with PeptiBAC prepared as previously described by complexing 1.5–6×10^6^ CFU of BCG with 40 nmol of CPP-OVA peptide (GRKKRRQRRRPQRWEKISIINFEKL) or no peptides. After 24 hours, cells were collected by scraping and stained with antigen-presenting cell (APC)-conjugated anti-mouse H-2K^b^ bound to SIINFEKL (141606, BioLegend), PerCP-conjugated anti-mouse CD86 (105025, BioLegend) and FITC-conjugated anti-mouse CD40 (124607, BioLegend) antibodies and analyzed by flow cytometry.

### Bacterial viability and macrophage assays

For the assessment of viability of the bacteria, CPP-containing peptide or poly-lysine-containing peptide was complexed with BCG (as described in the PeptiBAC complex formation-section) and complexes were directly plated for colony formation. Bacterial colonies were counted after 4 weeks of incubation at 37°C.

Mouse RAW-Blue macrophage reporter cell line (InvivoGen) expressing multiple pattern-recognition receptors (PRRs), including toll-like receptors (TLRs), NOD-like receptors (NLRs), RIG-I-like receptors (RLRs) and C-type lectin receptors (CLRs) was used to assess the activation of nuclear factor kappa-light-chain-enhancer of activated B cells (NF-kB) and activator protein 1 (AP-1) pathways induced by BCG and PeptiBAC. The presence of agonists of PRRs expressed by the RAW-Blue cells induces the activation of NF-kB and AP-1 leading to the secretion of embryonic alkaline phosphatase enzyme (SEAP). The substrate in the Quanti-BLUE (InvivoGen) system turns purple/blue in the presence of SEAP. The concentration of SEAP was measured using a multi-well plate reader (Varioskan Flash; ThermoLabsystems) to determine the relative activation efficacy of BCG and PeptiBAC. For the generation of bone marrow–derived macrophages (BMDMs), 10^7^ bone marrow cells isolated from C57BL/6JOlaHsd mouse were seeded in 10 mL of complete medium (RPMI-1640) (Sigma) containing 10 ng/mL recombinant macrophage colony-stimulating factor (Thermo Scientific), 10% FBS (Life Technologies), 2 mM L-glutamine, 50 U/mL penicillin, and 50 µg/mL streptomycin (Life Technologies). Cells were cultured at 37°C in a humidified atmosphere of 5% CO_2_. On day 3, half of the medium was replaced with fresh media. On day 6, part of the macrophages were harvested and used for cross-presentation experiments. For the rest of the macrophages, the media was gently aspirated and replaced with 10 mL of fresh complete medium containing 20 ng/mL interleukin-4 (IL-4, Life Technologies). Following 48 hours of culture, M2 polarized macrophages were harvested and used for polarization experiments.

### Animal experiments

All animal experiments were reviewed and approved by the Experimental Animal Committee of the University of Helsinki and the Provincial Government of Southern Finland (license number ESAVI/11895/2019). Animals were kept in individually ventilated cages under standard conditions (12 hours light:dark, temperature-controlled and humidity-controlled conditions) and received ad libitum access to water and food. Animals were monitored daily for symptoms related to distress and pain including hunched posture, overall activity/ability to move and roughness of the hair coat. Tumor dimensions were measured by caliper (largest tumor diameter and perpendicular tumor diameter) every second day, starting on the day tumors were first treated. All injections and tumor measurements were performed under isoflurane anesthesia.

For the B16-OVA melanoma experiment, 8-week to 9-week-old immunocompetent female C57BL/6JOlaHsd mice were injected in the right flank with 350,000 B16.OVA melanoma cells, and were treated 12, 15 and 22 days post tumor implantation with 0.75–3×10^5^ CFU/dose of BCG alone, 0.75–3×10^5^ CFU/dose of PeptiBAC-OVA, peptides alone or PBS as a mock-treated group. On day 27 post tumor implantation, 3 mice from each group were sacrificed, and spleens and tumors were collected for enzyme-linked immunospot (ELISPOT) and flow cytometry analysis. The remaining animals were followed up for survival.

For the B16F10.9/K1 melanoma experiment, 8-week to 9-week-old immunocompetent female C57BL/6JOlaHsd mice were injected in the right flank with 300,000 B16F10.9/K1 cells together with a 1:1 ratio of Matrigel Basement Membrane Matrix High Concentration (Corning, USA) and were treated 8, 10, and 22 days post tumor implantation with 6.25×10^6^–12×10^7^ CFU/dose of BCG, 6.25×10^6^–12×10^7^ CFU/dose of PeptiBAC-Trp2 or PBS as a mock-treated group. Groups receiving anti-PD-1 (InVivoMab, USA, clone RMP1-14) were injected intraperitoneally three times per week with 100 µg/dose starting at day 16 post tumor implantation.

For the CT26 colon experiment, 8-week to 9-week-old immunocompetent female BALB/c mice were injected in the right flank with 600,000 CT26 cells, and were treated 11, 13, and 25 days post tumor implantation with 6.25×10^6^–12×10^7^ CFU/dose of BCG, 6.25×10^6^–12×10^7^ CFU/dose of PeptiBAC-AH1 or PBS as a mock-treated group. Groups receiving anti-PD-1 (InVivoMab, USA, clone RMP1-14) were injected intraperitoneally three times per week with 100 µg/dose starting at day 17 post tumor implantation.

For the prime-boost vaccination experiments, 8-week to 9-week-old immunocompetent naïve female C57BL/6JOlaHsd mice were treated subcutaneously with 1×10^9^ VP/dose of PeptiCRAd VALO-mD901-Trp2, PeptiCRAd VALO-mD901-OVA, 2–8×10^6^ CFU/dose of PeptiBAC-Trp2, 2–8×10^6^ CFU/dose of PeptiBAC-OVA or saline as a mock-treated group. Vaccinations were performed 14 days apart. Four days after the last injection, mice were sacrificed and spleens were collected for ELISPOT assay. All mice strains were obtained from Envigo (Venray, the Netherlands).

### Flow cytometry

The following antibodies were used in the experiments: TruStain FcX anti-mouse CD16/32 (101320, BioLegend), FITC anti-mouse CD8 (A502-3B-E, ProImmune), Phycoerythrin (PE) anti-mouse CD3e (550353, BD Pharmingen), Peridinin-Chlorophyll-Protein (PerCP) anti-mouse CD19 (115531, BioLegend) and PE-Cyanine 7 anti-mouse CD4 (25-0041-82 eBioscience). SIINFEKL epitope-specific T cells were studied using APC-labeled H-2Kb/SIINFEKL pentamer (F093-84B-E, ProImmune). SVYDFFVWL (Trp2) epitope-specific T cells were studied using PE-labeled H-2Kb/SVYDFFVWL pentamer (F185-82B-E, Proimmune). SPSYVYHQF (AH1) epitope-specific T cells were studied using PE-labeled H-2Ld/SPSYVYHQF pentamer (F398-82A-E, Proimmune). Flow cytometric analysis were performed using a BD Accuri 6C Plus (BD Biosciences) or a BD LSRFortessa (BD Biosciences) flow cytometer and FlowJo software V.10 (BD Biosciences) was used for data analysis (see online supplemental figure 7 for gating strategies used in the experiments).

10.1136/jitc-2021-002707.supp2 Supplementary data

### ELISPOT assays

The amount of SIINFEKL (OVA_257-264_), SVYDFFVWL (TRP2_180-188_), BCG and adenovirus-specific activated, interferon-γ secreting T cells were measured by ELISPOT assay (CTL, Ohio, USA) according to the manufacturer’s instructions. Briefly, 2 µg of SIINFEKL or SVYDFFVWL peptide was used to stimulate the antigen-presenting cells (APCs). After 2 or 3 days of stimulation, plates where stained and sent to CTL-Europe GmbH for counting of the spots.

### Statistical analysis

Statistical analysis was performed using GraphPad Prism V.8.0 software (GraphPad Software, USA). For data analysis, one-way analysis of variance was used. All results are expressed as mean±SEM.

## Results

### BCG can be coated with therapeutic peptides by using a cell penetrating peptide sequence or a poly-lysine sequence as an anchor

**Figure 1:**
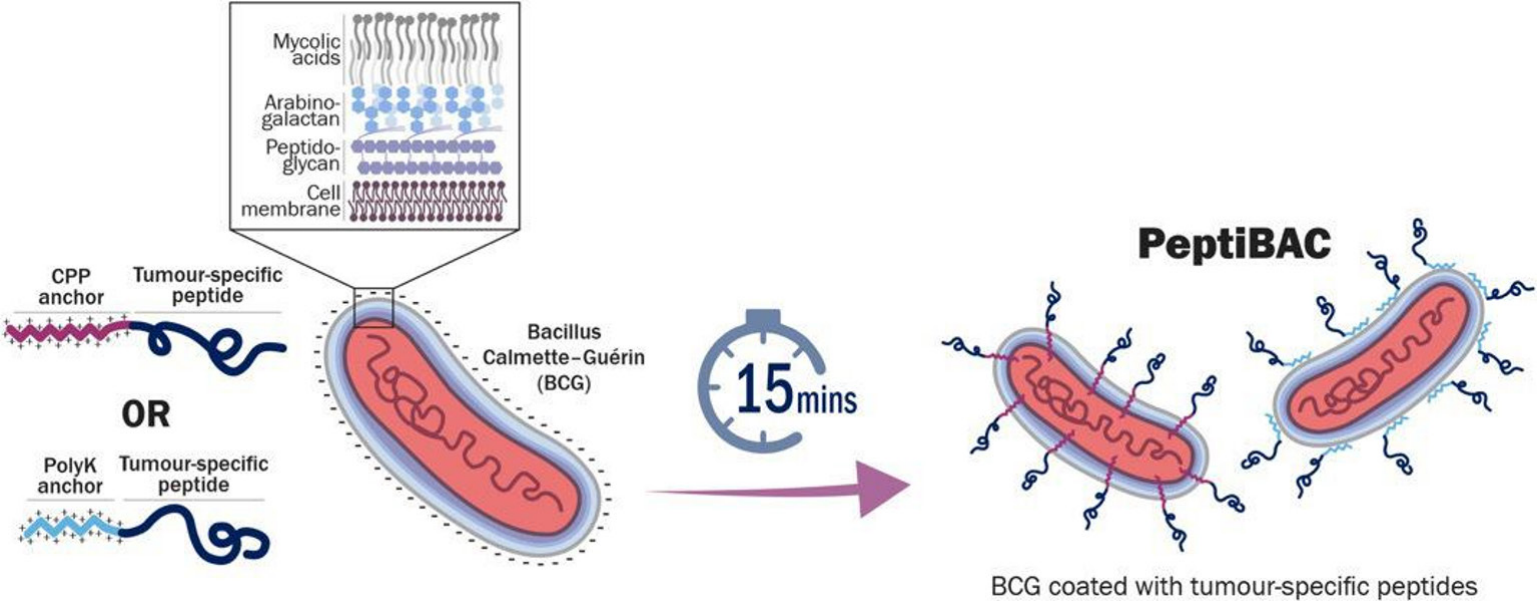
A schematic presentation of a PeptiBAC cancer vaccine platform. Tumor antigens can readily be attached to the mycobacterial outer membrane of BCG using a cell penetrating peptide (CPP) sequence or a poly-lysine sequence as an anchoring moiety. Anchor-modified peptides are complexed for 15 min with BCG for efficient attachment. Unbound peptides are removed by pelleting the bacteria followed by buffer exchange. Various different peptides, including MHC class I and II epitopes, can be delivered by the PeptiBAC platform for potent activation of antigen-presenting cells and increased antigen-specific immunological responses. MHC, major histocompatibility complex; PeptiBAC, peptide-coated Bacillus Calmette-Guérin.

**Figure 2:**
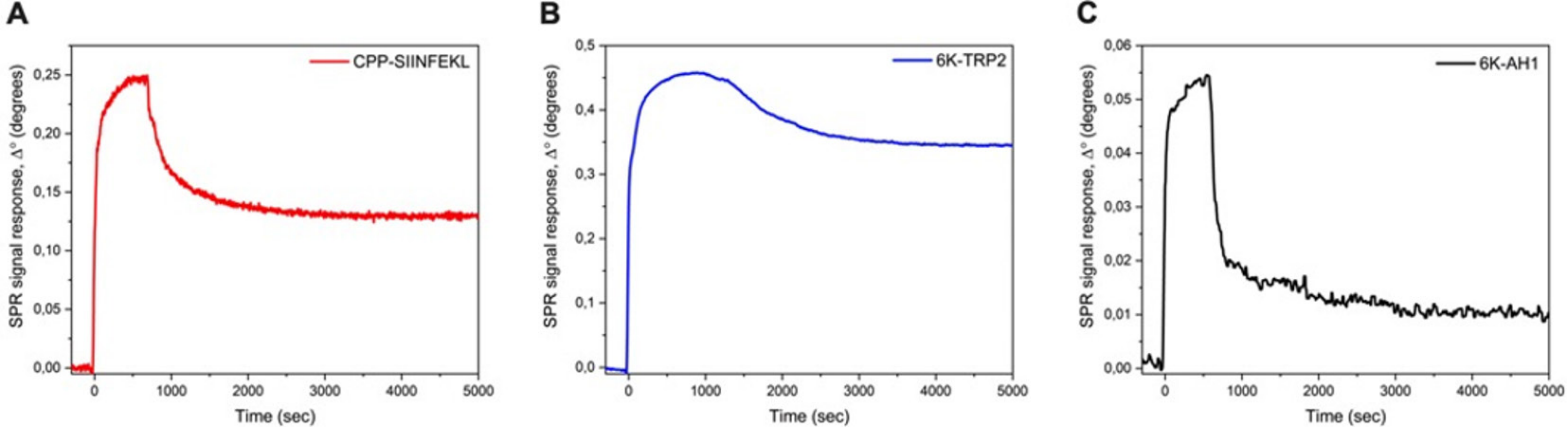
Surface plasmon resonance (SPR) analysis of the peptide/BCG interaction. (A) SPR analysis of the interaction between the CPP-OVA and BCG. (B) SPR analysis of the interaction between the polyK-Trp2 and BCG. (C) SPR analysis of the interaction between the polyK-AH1 and BCG. CPP, cell penetrating peptide; OVA, ovalbumin.

The mycobacterial cell wall is a highly complex structure containing multiple layers of different lipid components and has an extremely negative surface potential.^19–21^ We hypothesized that therapeutic peptide sequences could be attached into the mycobacterial cell wall using a cell penetrating peptide (CPP) sequence or a poly-lysine sequence as attachment moieties (see figure 1 for schematic presentation of the PeptiBAC platform). Various CPP sequences were tested by SPR for their efficacy at anchoring therapeutic peptides into the mycobacterial cell wall (see online supplemental figure 1), and a CPP sequence derived from HIV Tat protein was found to be the most efficient CPP sequence for anchoring the peptides (figure 2A). In addition to the CPP sequence derived from HIV Tat, a positively charged poly-lysine sequence was found to efficiently anchor the peptides into the cell wall (figure 2B, C). We estimated the number of peptides bound to BCG bacterium using these two different attachment moieties. For the SIINFEKL antigen containing an N-terminal CPP Tat sequence, the number of peptides bound to BCG was estimated to be 1.8×10^6^ peptide molecules/bacterium. For the Trp2 antigen and for the AH1 antigen containing N-terminal poly-lysine sequences, the number of peptides bound to BCG was estimated to be 4.4×10^6^ peptide molecules/bacterium and 3.2×10^5^ peptide molecules/bacterium, respectively.

10.1136/jitc-2021-002707.supp3 Supplementary data

### APCs can efficiently present therapeutic peptides delivered by PeptiBAC

**Figure 3:**
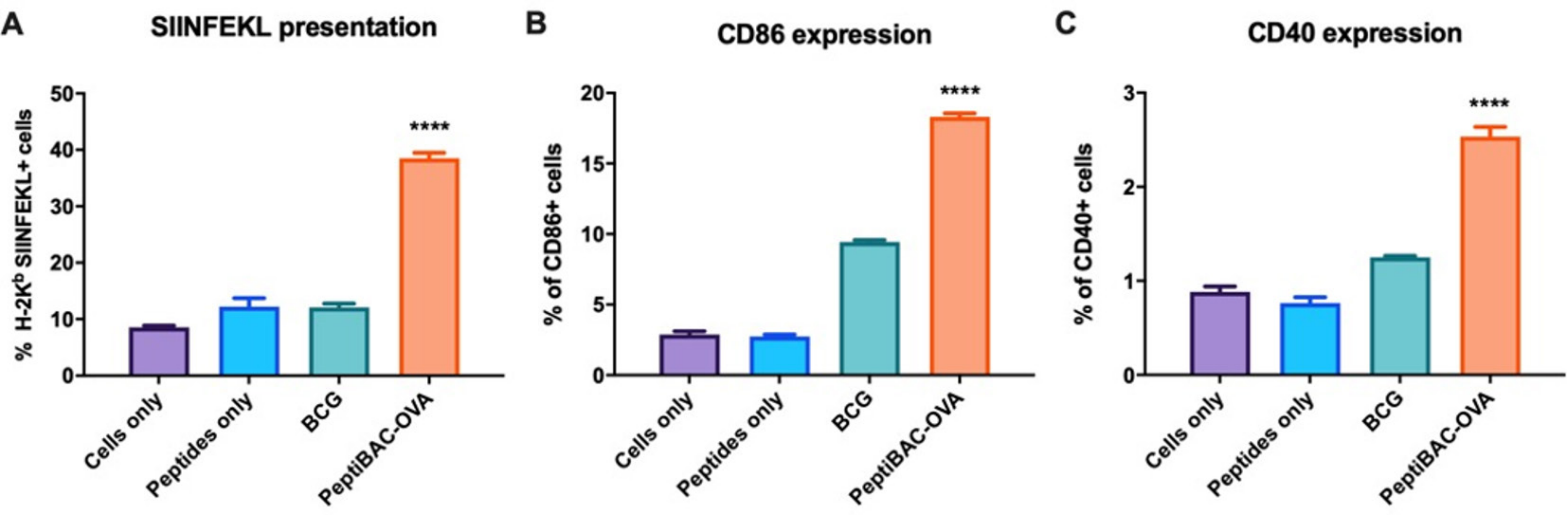
Antigen-presenting cells can readily cross-present antigens delivered by the PeptiBAC platform. Mouse dendritic cell line Jaws II was pulsed with PeptiBAC-OVA, BCG, CPP-containing SIINFEKL peptide alone or left unpulsed (cells only). Cross-presentation was determined by flow cytometry using APC-conjugated anti-H-2Kb bound to SIINFEKL. CD86 and CD40 expression (as a measure of dendritic cell maturation and activation) was determined by flow cytometry. Each bar is the mean±SEM of technical triplicates. Statistical analysis was performed with one-way analysis of variance. ****p<0.0001. APC, antigen-presenting cell; CPP, cell penetrating peptide; PeptiBAC, peptide-coated Bacillus Calmette-Guérin; PeptiBAC-OVA, OVA-targeting PeptiBAC.

Next, we tested whether the PeptiBAC platform can deliver therapeutic peptides to APCs and if the APCs can cross-present the MHC-I epitope portions from these peptides. PeptiBAC-OVA (BCG coated with CPP-containing immunodominant epitope from chicken ovalbumin; GRKKRRQRRRPQRWEKISIINFEKL) was used to infect JAWSII murine DCs for 24 hours followed by the assessment of the cross-presentation efficacy of the epitope (SIINFEKL) by flow cytometry (figure 3A). As expected, PeptiBAC-delivered SIINFEKL was efficiently cross-presented by the DCs, as almost 40% of JAWSII cells were shown to cross-present the SIINFEKL epitope. In addition, PeptiBAC-OVA was able to induce enhanced DC activation compared with BCG, as assessed by the significantly increased expression of cluster of differentiation 86 and 40 (CD86 and CD40) proteins (figure 3B, C, respectively).

### Intratumoral treatment with PeptiBAC with CPP-containing OVA antigen induces systemic tumor-specific CD8^+^ T cell response in syngeneic mouse model of B16.OVA melanoma

**Figure 4:**
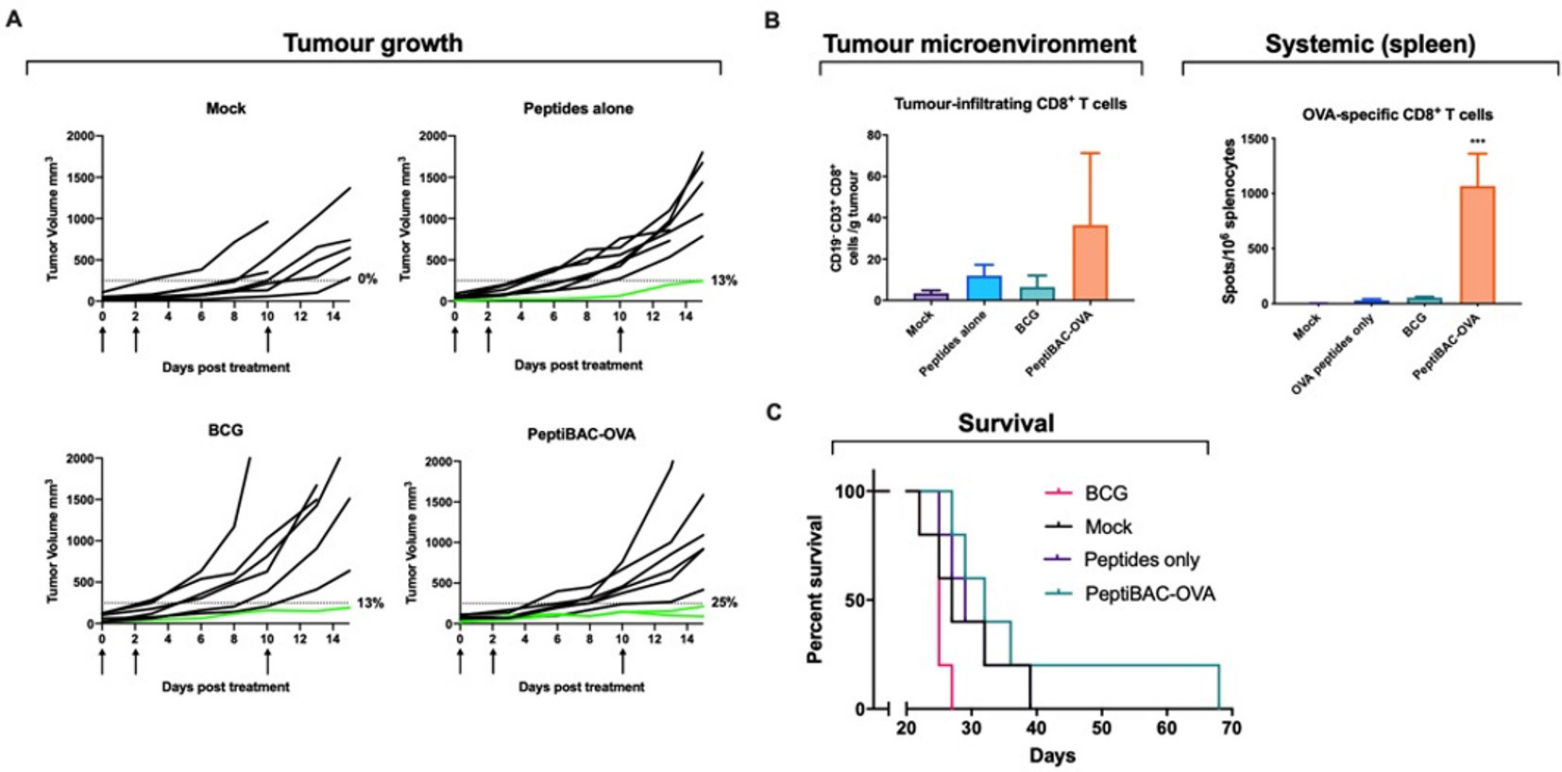
Intratumorally administered PeptiBAC induces systemic tumor-specific T cell responses in a syngeneic mouse model of B16.OVA melanoma. (A) BCG, peptides alone or PeptiBAC-OVA was given intratumorally 12, 15 and 22 days post tumor implantation. Individual tumor growth curves for all treatment groups are shown. A threshold of 250 mm^3^ was set to define the percentage of mice responding to the different therapies (dotted line). The percentage of responders in each treatment group is shown on the right side of the dotted line. (B) Immunological analysis of tumors and spleens of treated mice. (C) Kaplan-Meier survival curve for the treatment groups. The number of mice in each group was 7–8. Statistical analysis was performed with one-way analysis of variance. ***p<0.001. OVA, ovalbumin; PeptiBAC, peptide-coated Bacillus Calmette-Guérin; PeptiBAC-OVA, OVA-targeting PeptiBAC.

To study the immunostimulatory potential and antitumor effects of the PeptiBAC platform, we used a well-established syngeneic mouse melanoma model B16 expressing chicken ovalbumin (OVA) as a model antigen.^22^ When mice-bearing B16.OVA tumors were treated intratumorally with OVA-targeting PeptiBAC (PeptiBAC-OVA), BCG, peptides alone or vehicle (mock), we observed a modest and non-significant increase in tumor growth control in the PeptiBAC-OVA group as compared with other treatment groups (figure 4A). We set a tumor size threshold of 250 mm^3^ for defining the responders in each treatment group. In mock-treated group, there were no responders, while groups treated with the CPP-containing SIINFEKL peptide alone or BCG, both had one mouse defined as a responder to the therapy. PeptiBAC-OVA treatment had only modest effect on tumor growth with two mice defined as responders for the therapy; a 25% response rate for this group of mice. We went on to analyze whether there were any differences in immunological responses against the OVA antigen between the treatment groups, and we first assessed whether there were any differences in the infiltration of immune cells into the TME. We observed that a higher (although non-significant) number of cytotoxic CD8^+^ T cells infiltrated into the tumors of PeptiBAC-OVA-treated mice as compared with the tumors of BCG-treated, peptide alone-treated or mock-treated mice. However, we did not see any infiltration of tumor-specific CD8^+^ T cell into the tumors in any of the treatment groups (data not shown). In contrast to BCG-treated, peptide alone-treated and mock-treated mice, a significant induction of a systemic OVA-specific T cell response was seen in PeptiBAC-OVA-treated mice (figure 4B). The modest increase in tumor growth control in the PeptiBAC-OVA group translated into a non-significant trend toward longer survival, with median survival of 32 days compared with 25, 29 and 27 days with BCG, peptide alone and mock groups, respectively (figure 4C).

### CPP-containing but not poly-lysine-containing antigenic peptides reduce the viability of BCG

The unexpected minimal efficacy seen using PeptiBAC with CPP-containing OVA antigen prompted us to test whether the CPP-containing antigen peptide could be toxic to the bacteria. Indeed, we saw a decrease in BCG viability when coated with CPP-containing antigen peptide but not when coated with poly-lysine-containing antigen peptide. To further validate the poly-lysine as a suitable attachment moiety, we tested macrophage activation potential of PeptiBAC coated with poly-lysine containing antigen peptide. PeptiBAC with poly-lysine-containing antigen peptide was equally potent in activating NF-kB/AP1 pathways in murine RAW-blue macrophages as the non-coated BCG (online supplemental figure 2). As the tumor-associated macrophages (TAMs) are an important cell component of the TME, we also wanted to assess the cross-presentation properties of macrophages on PeptiBAC-delivered tumor antigens. PeptiBAC-OVA (BCG coated with poly-lysine-containing OVA peptide was used to infect BMDMs for 24 hours followed by the assessment of the cross-presentation efficacy of the epitope (SIINFEKL) by flow cytometry. Remarkably, PeptiBAC-delivered SIINFEKL was efficiently cross-presented on the surface of the BMDMs (online supplemental figure 3A). In addition to macrophage presentation, we wanted to see whether PeptiBAC had the same properties as BCG on macrophage polarization from M2 state more toward the M1 state. M2 polarized macrophages were infected with BCG or PeptiBAC, and the expressions of macrophage M2 and M1 markers were analyzed by flow cytometry. Both BCG and PeptiBAC were equally effective at polarizing M2 macrophages more toward the M1 state as assessed by the significant upregulation of both MHC-II and CD86 expression and by the significant downregulation of the M2 marker CD206 expression (online supplemental figure 3B). Based on these data, poly-lysine was chosen as the attachment moiety to be used in all further experiments.

10.1136/jitc-2021-002707.supp4 Supplementary data

10.1136/jitc-2021-002707.supp5 Supplementary data

### Intratumoral treatment with PeptiBAC with poly-lysine-containing Trp2 antigen increases the number of responders to anti-PD-1 therapy, improves tumor control and induces tumor-specific T cell responses in a syngeneic mouse model of B16.F10.9/K1 melanoma

**Figure 5:**
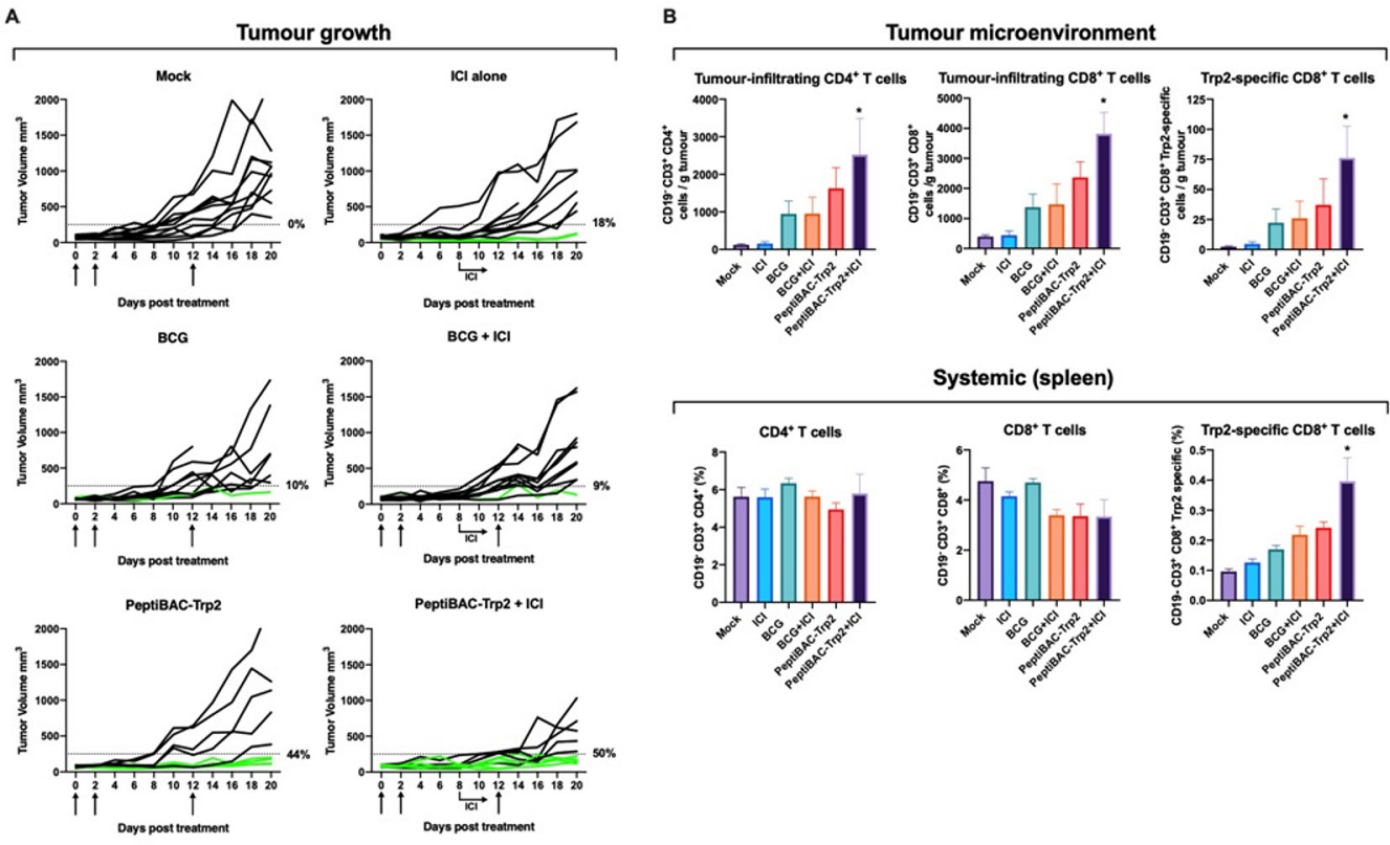
PeptiBAC in combination with anti-PD1 improves tumor growth control compared with either monotherapy and induces robust infiltration of tumor-specific CD8^+^ T cells into tumors in a syngeneic mouse model of B16.F10.9/K1 melanoma. (A) Anti-PD-1 immune checkpoint inhibitor (ICI) alone (100 µg/dose given intraperitoneally three times a week, starting at day 8), BCG alone or in combination with anti-PD-1 ICI and PeptiBAC-Trp2 alone or in combination with anti-PD-1 ICI was given intratumorally 8, 10, and 22 days post tumor implantation. Individual tumor growth curves for all treatment groups are shown. A threshold of 250 mm^3^ was set to define the percentage of mice responding to the different therapies (dotted line). The percentage of responders in each treatment group is shown on the right side of the dotted line. (B) Immunological analysis of tumors and spleens of treated mice. The number of mice in each group was 9–11. Statistical analysis was performed with one-way analysis of variance. *p<0.05. PeptiBAC, peptide-coated Bacillus Calmette-Guérin; PD-1, programmed death 1.

Next, we tested the PeptiBAC platform in a syngeneic mouse model of B16.F10.9/K1 melanoma using a more relevant, tumor-associated antigen from tyrosinase-related protein 2 (Trp2_180–188_) in combination with anti-PD-1 ICI therapy. B16.F10.9/K1 melanoma is a derivative of a highly metastatic B16.F10.9 melanoma with a low cell surface expression of major histocompatibility complex 1 (MHC-I) H-2Kb that was transfected with H-2Kb genes to generate H-2Kb-expressing clone K1.^23^ The B16.F10.9/K1 clone is more responsive to cancer immunotherapies than the highly immunosuppressive parental strain B16.F10.9. Starting at 8 days post tumor engraftment, mice were treated intratumorally with BCG, anti-PD-1 alone, PeptiBAC-Trp2, BCG in combination with anti-PD-1, PeptiBAC-Trp2 in combination with anti-PD-1 or saline as a mock-treated group. Again, we set the tumor size threshold of 250 mm^3^ for defining the responders in each treatment group. In contrast to mock-treated animals, BCG, anti-PD-1 alone and BCG in combination with anti-PD-1 ICI-treated groups showed modest tumor growth control with response rates of 10%, 18% and 9%, respectively. PeptiBAC-Trp2-treated animals showed robust tumor growth control with a 44% response rate. Remarkably, PeptiBAC-Trp2 in combination with anti-PD-1-treated animals showed efficient tumor growth control, with 50% response rate; increasing the response rate for anti-PD-1 therapy from 18% to 50% (figure 5A, see also online supplemental figure 4 for average tumor growth curves). To further evaluate the mechanism of tumor growth control, we assessed whether there were any differences in the Trp2-specific T cell responses between the treatment groups. We saw a non-significant trend toward increased numbers of tumor-infiltrating CD4^+^ and CD8^+^ T cells in PeptiBAC-Trp2-treated tumors compared with BCG, anti-PD-1 alone and BCG in combination with anti-PD-1 ICI-treated tumors. Also, we saw a non-significant trend toward increased number of Trp2-specific CD8^+^ T cells in PeptiBAC-Trp2-treated tumors compared with BCG, anti-PD-1 alone and BCG in combination with anti-PD-1 ICI-treated tumors. In contrast to other treatment groups, PeptiBAC-Trp2 in combination with anti-PD-1-treated tumors had significantly more tumor-infiltrating CD4^+^ and CD8^+^ T cells as well as Trp2-specific CD8^+^ T cells, indicating an enhanced effect on T cell responses by combining the two treatment modalities (figure 5B, upper panel). We also evaluated systemic tumor-specific T cell responses by analyzing the spleens of treated mice. No significant differences in the number of CD4^+^ and CD8^+^ T cells were found between groups. The number of Trp2-specific CD8^+^ T cells was increased in PeptiBAC-Trp2 in combination with anti-PD-1 ICI-treated spleens as compared with other treatment groups, again indicating an enhanced effect on T cell responses by combining the two treatment modalities (figure 5B, lower panel).

10.1136/jitc-2021-002707.supp6 Supplementary data

### Intratumoral treatment with PeptiBAC with poly-lysine-containing modified gp70 antigen increases the number of responders to anti-PD-1 therapy, improves tumor control and induces tumor-specific T cell responses in a syngeneic mouse model of CT26 colorectal cancer

**Figure 6:**
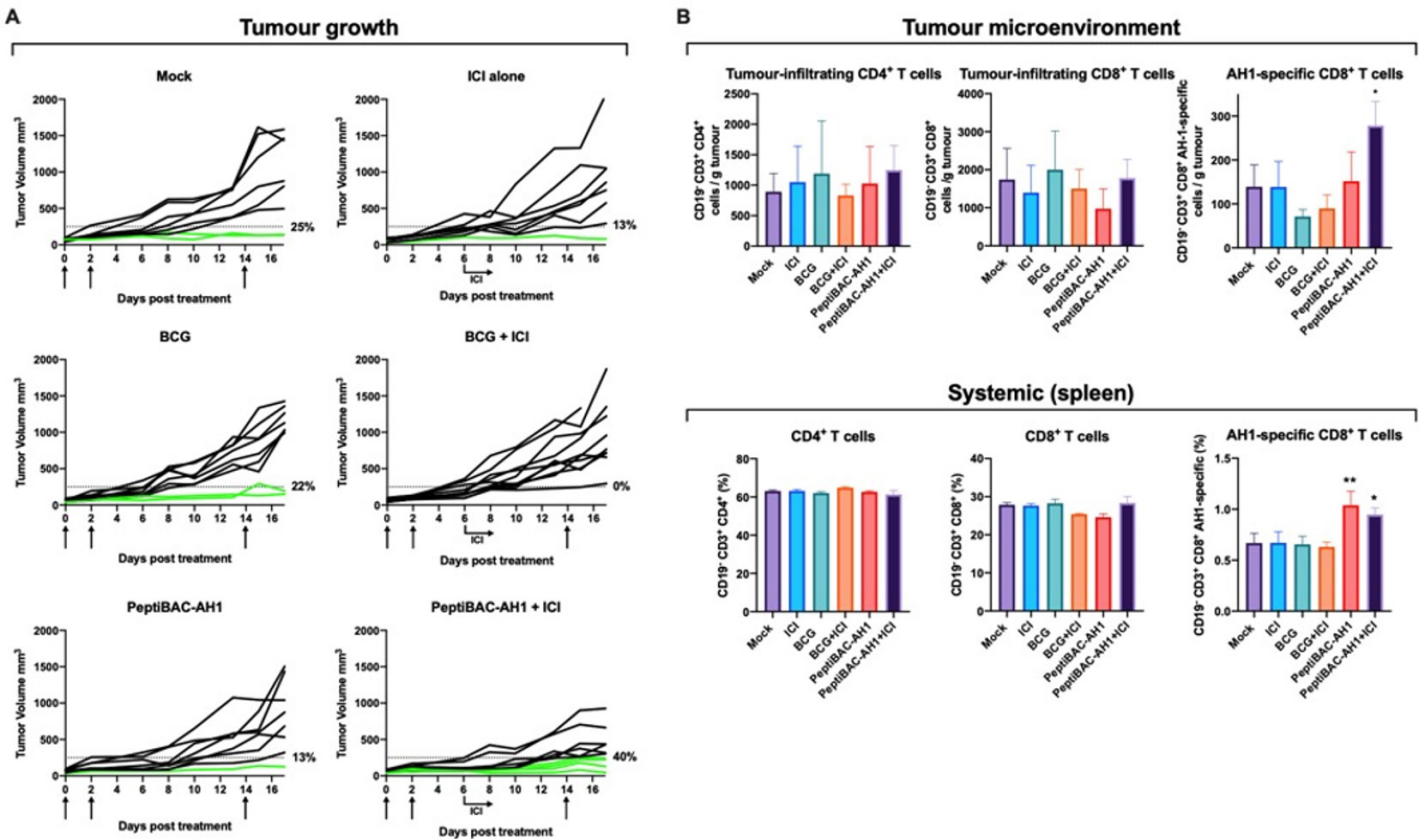
PeptiBAC in combination with anti-PD1 improves tumor growth control compared with either monotherapy and induces systemic tumor-specific CD8^+^ T cell responses and robust infiltration of tumor-specific CD8^+^ T cells into the tumor in a syngeneic mouse model of CT26 colorectal cancer. (A) Anti-PD-1 immune checkpoint inhibitor (ICI) alone (100 µg/dose given intraperitoneally three times a week, starting at day 6), BCG alone or in combination with anti-PD-1 ICI and PeptiBAC-AH1 alone or in combination with anti-PD-1 ICI was given intratumorally 11, 13, and 25 days post tumor implantation. Individual tumor growth curves for all treatment groups are shown. A threshold of 250 mm^3^ was set to define the percentage of mice responding to the different therapies (dotted line). The percentage of responders in each treatment group is shown on the right side of the dotted line. (B) Immunological analysis of tumors and spleens of treated mice. The number of mice in each group was 8–10. Statistical analysis was performed with one-way analysis of variance: *p<0.05; **p<0.01. PeptiBAC, peptide-coated Bacillus Calmette-Guérin; PD-1, programmed death 1.

To validate the PeptiBAC platform as a more universal cancer vaccine platform, we tested the platform in a syngeneic mouse model of CT26 colorectal cancer using a modified tumor rejection antigen AH1 in combination with anti-PD-1 ICI therapy. AH1 represents one of the best characterized tumor rejection antigens in mice and is derived from the gp70 envelope protein of murine leukemia virus (MuLV), which is endogenous in the genome of most laboratory mouse strains, including the BALB/c strain used in these studies.^24^ Starting at 11 days post tumor engraftment, mice were treated intratumorally with BCG, anti-PD-1 alone, PeptiBAC-AH1, BCG in combination with anti-PD-1, PeptiBAC-AH1 in combination with anti-PD-1 or saline as a mock-treated group. Once again, the tumor size threshold was set to 250 mm^3^ for defining the responders in each treatment group. Mock, BCG, anti-PD-1 alone and BCG in combination with anti-PD-1 ICI-treated groups showed tumor growth characteristics with response rates of 25%, 22%, 13% and 0%, respectively. Interestingly, in contrast to the B16.F10.9/K1 melanoma model, PeptiBAC-AH1 treatment alone did not increase tumor growth control relative to the other groups, with a response rate of only 13%. Strikingly, PeptiBAC-AH1 in combination with anti-PD-1-treated animals showed efficient tumor growth control with a 40% response rate; increasing the response rate for anti-PD-1 therapy from 13% to 40% (figure 6A, see also online supplemental figure 5 for average tumor growth curves). Again, we assessed whether there were any differences in T cell responses between the treatment groups. We saw no significant differences in the numbers of tumor-infiltrating CD4^+^ and CD8^+^ T cells between the treatment groups, although, interestingly, the number of CD8^+^ T cells in the PeptiBAC-AH1-treated tumors was slightly but non-significantly decreased compared with tumors from other treatment groups. While the number of AH1-specific CD8^+^ T cells was slightly but non-significantly decreased in BCG and BCG in combination with anti-PD-1 ICI-treated tumors when compared with the mock group, PeptiBAC-AH1 in combination with anti-PD-1 ICI-treated tumors had significantly increased numbers of AH1-specific CD8^+^ T cells, suggesting a correlation between tumor growth control and the number of AH1-specific CD8^+^ T cells in the TME (figure 6B, upper panel). Analysis of systemic tumor-specific T cell responses from the spleens of the treated mice showed no significant differences in the number of CD4^+^ and CD8^+^ T cells between groups. However, a significant increase in AH1-specific CD8^+^ T cells was seen in the PeptiBAC-AH1 and PeptiBAC-AH1 in combination with anti-PD-1 ICI-treated mice spleens as compared with spleens from other groups (figure 6B, lower panel).

10.1136/jitc-2021-002707.supp7 Supplementary data

### Heterologous prime-boost vaccination strategy combining PeptiBAC platform with PeptiCRAd platform improves T cell responses against the coated antigen

**Figure 7:**
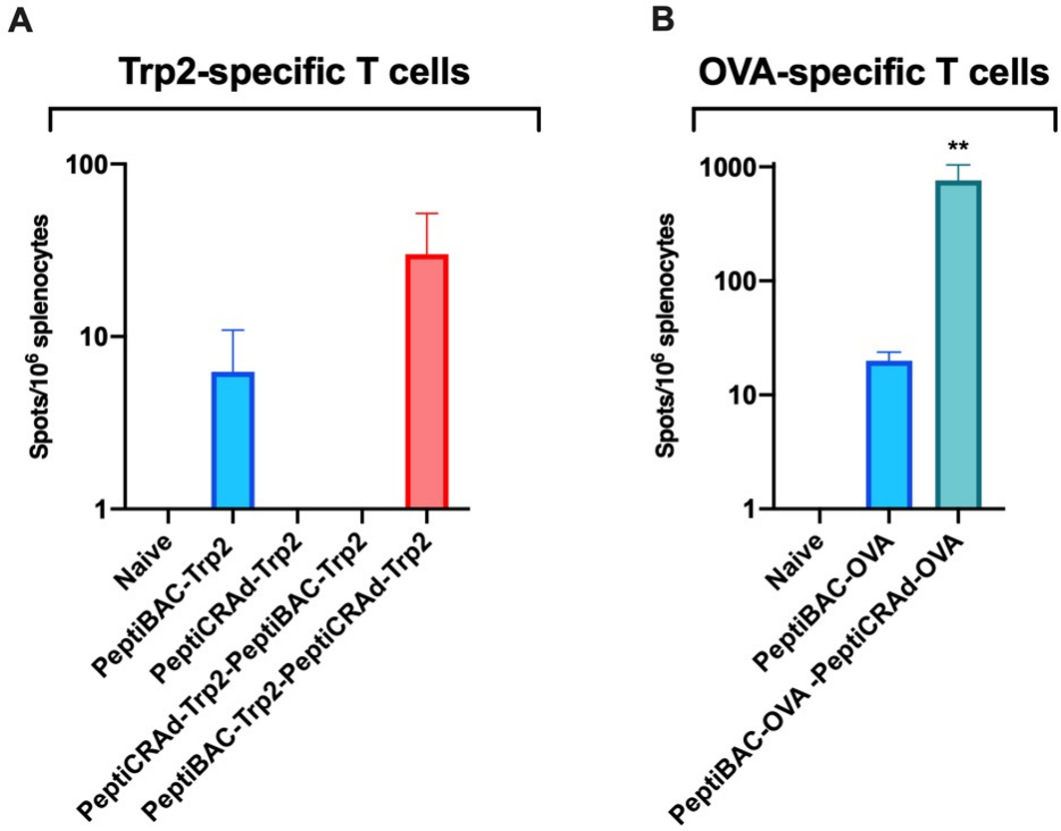
Heterologous prime-boost vaccination with PeptiCRAd platform improves peptide-specific T cell responses elicited by the PeptiBAC platform. (A) Naïve C57BL/6JOlaHsd immunocompetent mice were vaccinated subcutaneously with 1×10^9^ VP/dose of PeptiCRAd-Trp2 or 2–8×10^6^ CFU/dose of PeptiBAC-Trp2 or saline as a mock-treated group. Prime and boost vaccinations were performed 14 days apart, and 4 days after the boost, mice were sacrificed, and spleens were collected for enzyme-linked immunospot (ELISPOT) assay. The number of mice in each vaccination group was 4, and in control group not receiving vaccinations the number of mice was 2. (B) Similar to A, mice were vaccinated with PeptiBAC-OVA or PeptiBAC-OVA followed by PeptiCRAd-OVA booster. The number of mice in each vaccination group was 5. OVA, ovalbumin; PeptiBAC, peptide-coated Bacillus Calmette-Guérin; PeptiBAC-OVA, OVA-targeting PeptiBAC; PeptiCRAd, peptide-coated conditionally replicating adenovirus.

Finally, the PeptiBAC-platform was tested in combination with our recently described cancer vaccine platform PeptiCRAd^14^ (**pepti**de-coated **c**onditionally **r**eplicating **ad**enovirus) using a heterologous prime-boost vaccination strategy. The adenovirus used in the PeptiCRAd platform was an adenovirus serotype 5 expressing murine CD40L and OX40L. By combining two immunologically distinct platforms coated with the same antigen, we tested whether this heterologous prime-boost approach could enhance T cell-specific immune responses in naïve mice toward the MHC-I restricted epitope presented by both platforms. To this end, we vaccinated naïve C57BL/6JOlaHsd mice with two doses of PeptiBAC-Trp2 or PeptiCRAd-Trp2 as homologous prime-boost controls or with PeptiBAC-Trp2 prime followed by PeptiCRAd-Trp2 boost and PeptiCRAd-Trp2 prime followed by PeptiBAC-Trp2 boost with doses given 14 days apart. Four days after the boost dose, mice where sacrificed and the spleens were harvested and analyzed for the induction of Trp2-specific T cell responses by interferon-gamma ELISPOT. Vaccination with PeptiCRAd-Trp2 homologous prime-boost or PeptiCRAd-Trp2–PeptiBAC-Trp2 heterologous prime-boost did not induce Trp2-specific T cell responses in this vaccination setting. PeptiBAC-Trp2 homologous prime-boost vaccination induced moderate Trp2-specific T cell responses which were enhanced by the PeptiBAC-Trp2–PeptiCRAd-Trp2 heterologous prime-boost vaccination regimen (figure 7A). Subsequently, we tested the same approach using the immunodominant epitope of ovalbumin (SIINFEKL), an epitope more immunogenic than Trp2, and assessed the induction of OVA-specific T cell responses again by using the interferon-gamma ELISPOT. Here, the PeptiBAC-OVA–PeptiCRAd-OVA heterologous prime-boost regimen induced significant enhancement of OVA-specific T cell responses compared with PeptiBAC-OVA vaccination (figure 7B).

## Discussion

In this study, we have shown that by coating the mycobacterial outer membrane of BCG with MHC class I-restricted tumor-associated epitopes, we were able to broaden the immune responses elicited by the bacteria to include the coated antigens. As the attachment moiety for coating the therapeutic peptides onto the mycobacterial outer membrane, we tested both the CPP sequence of the HIV Tat protein fused to the N terminus of the tumor epitopes and a stretch of 6 lysine residues similarly fused to the N terminus of the tumor epitopes. We have previously shown that the CPP sequence and the poly-lysine sequence at the N-terminus of the therapeutic peptides do not influence the presentation of the tumor epitopes from these peptides by APCs.^14 25^ Both attachment moieties were able to efficiently attach therapeutic peptides onto the mycobacterial outer membrane, and BCG coated with an immunodominant epitope derived from chicken ovalbumin (PeptiBAC-OVA) was able to deliver these peptides into APCs followed by efficient processing and presentation by the APCs. The antitumor and immune-activating properties of PeptiBAC-OVA were tested in a syngeneic mouse model of B16.OVA melanoma. Although PeptiBAC-OVA induced significant systemic OVA-specific T cell responses, the effect on tumor growth control was modest at best. In line with earlier reports,^26 27^ we did not observe any beneficial effect on tumor growth control by intratumoral treatment with BCG. Interestingly, while PeptiBAC-OVA-treated mice had the longest average survival, we observed a trend toward decreased survival with the BCG-treated group of mice. The minimal in vivo efficacy seen with PeptiBAC with CPP-containing OVA was most likely due to the toxic effects of the CPP-containing peptide coated onto the BCG. Indeed, we noticed a decrease in viability of the BCG after complexation with the CPP-containing OVA peptide. As poly-lysine sequence also enabled efficient coating of therapeutic peptides onto the mycobacterial outer membrane, we also tested the effects of poly-lysine-containing peptide on viability of BCG after complexation. Poly-lysine-containing peptide did not affect the viability of the BCG nor the NF-kB/AP1 pathway activation as assessed by using RAW-blue murine macrophage reporter cell line. Since TAMs are an integral cellular component of the TME, we also tested the ability of PeptiBAC to induce antigen cross-presentation on infection of macrophages. In addition, we assessed the capability of PeptiBAC to drive macrophage polarization from M2 toward more M1-like macrophages. Interestingly, macrophages were able to readily cross-present PeptiBAC-delivered antigens, and in addition, PeptiBAC was able to drive macrophage polarization from M2 more toward M1-like phenotype.

We next tested the efficacy of PeptiBAC complexed with poly-lysine-containing Trp2 epitope (PeptiBAC-Trp2) in combination with ICI therapy using an antibody against murine PD-1 in a syngeneic mouse model of B16.F10.9/K1 melanoma. In this model, monotherapy with PeptiBAC-Trp2 induced an increase in the number of mice responding to the therapy as compared with Mock, BCG, ICI or BCG+ICI-treated groups. Remarkably, PeptiBAC-Trp2 treatment efficiently sensitized tumors to ICI therapy and the combination therapy group showed a response rate of 50%. In addition to increased tumor growth control, immunological analysis of the treated tumors revealed significant infiltration of CD4^+^, CD8^+^ as well as Trp2-specific CD8^+^ T cells into the TME of the PeptiBAC-Trp2+ICI-treated mice.

To further evaluate the PeptiBAC platform, we tested the platform in a syngeneic mouse model of CT26 colorectal cancer using a modified tumor rejection antigen AH1 in combination with anti-PD-1 ICI therapy. In this model, although we did not see effects on tumor growth with either monotherapies, the combination of PeptiBAC-AH1 and anti-PD-1 ICI had enhanced antitumor effects, showing a response rate of 40%. In addition, the combo-treated mice showed significantly increased infiltration of AH1-specific CD8^+^ T cells into the TME. Both PeptiBAC-AH1 monotherapy and PeptiBAC-AH1 in combination with anti-PD-1 significantly increased AH1-specific CD8^+^ T cells in spleens as compared with other treatment groups.

Heterologous prime-boost vaccination sequentially using two or more immunologically distinct platforms to deliver the antigen(s) has previously been tested in both infectious disease and cancer settings,^28–32^ and has shown to be able to induce enhanced T cell responses against the antigen as compared with homologous prime-boost vaccination. Also, BCG has previously been used as a component in heterologous prime-boost settings.^33–35^ Here, we set out to test whether the PeptiBAC platform could be used as a component of a heterologous prime-boost vaccination setting together with another peptide-based cancer vaccine platform using oncolytic adenoviruses, called PeptiCRAd. Interestingly, we saw enhanced antigen-specific T cell responses as compared with homologous prime-boost vaccination with PeptiBAC only when PeptiBAC was used as a priming vaccine and PeptiCRAd as a booster vaccine. The adenovirus used in the PeptiCRAd platform was an adenovirus serotype 5 expressing murine CD40L and OX40L.

In addition to CIS, BCG is the preferred treatment for high-risk non-muscle-invasive bladder cancer (NMIBC) and an option for intermediate-risk NMIBC.^36^ Recently, the US Food and Drug Administration approved an ICI against PD-1 (pembrolizumab) to treat patients with BCG-unresponsive, high-risk, NMIBC with carcinoma in situ with or without papillary tumors who are ineligible for, or have elected not to undergo cystectomy.^37^ In addition, a recent phase III trial that evaluated a novel intravesical therapy, nadofaragene firadenovec (a non-replicating adenovirus vector expressing human IFNα2b) in 151 patients with BCG-unresponsive NMIBC reported that more than half of the patients achieved a complete response, of whom almost half maintained complete response at 12 months.^38^ It is intriguing to hypothesize, in light of the data presented here, that using PeptiBAC with tumor-specific (neo)antigens identified from bladder cancer to treat NMIBC could increase the response rate of BCG therapy, and in addition, if used in combination with pembrolizumab, could have significant improvements over outcomes achieved with BCG or pembrolizumab as monotherapies. Nadofaragene firadenovec is compatible with the PeptiCRAd cancer vaccine platform and could be tested as part of the PeptiCRAd platform together with prior therapy with PeptiBAC as a heterologous prime-boost cancer vaccine immunotherapy. Compared with various other immunotherapy approaches, the PeptiBAC platform is highly adaptable and can be quickly coated with a patient’s unique set of tumor-specific antigens, a prerequisite for personalized cancer immunotherapy. Most importantly, this platform could be transferred into the clinical setting very fast, since the backbone of the platform, the BCG vaccine, is already FDA/EMEA approved for cancer immunotherapy for bladder cancer and melanoma.

In addition to being used as a cancer immunotherapy, BCG is the only vaccine used in infants and neonates to prevent tuberculous meningitis and disseminated tuberculosis.^39^ Remarkably, in addition to its specific effect against tuberculosis, the BCG vaccine has beneficial non-specific (off-target) effects on the immune system that protect against a wide range of other infections, including bacteria like *Staphylococcus aureus*, fungi like *Candida albicans* and viruses like the yellow fever virus.^40 41^ Recent studies have suggested that countries that mandate BCG vaccination for the population have a lower number of infections and a reduced mortality from COVID-19.^42^ Based on these data, it has been hypothesized that BCG vaccination might be a potent preventive measure against SARS-CoV-2 infection and/or may reduce COVID-19 disease severity. Currently, there are at least nine clinical studies ongoing to determine the effect of BCG vaccination on outcomes from COVID-19. However, the efficacy of the BCG vaccine to provide protection against COVID-19 might be significantly improved by enhancing the SARS-CoV-2-specific cellular immune responses elicited by the BCG vaccine by the use of PeptiBAC platform with SARS-CoV-2-specific antigens.

## Data Availability

All data relevant to the study are included in the article and are available upon reasonable request (vincenzo.cerullo@helsinki.fi).
