# Supplementary material for: Novel personalized cancer vaccine platform based on Bacillus Calmette-Guèrin
Source: J Immunother Cancer. 2021 Jul 15;9(7):e002707. doi: 10.1136/jitc-2021-002707 (PMC8286790; doi:10.1136/jitc-2021-002707)
Supplement: Supplementary data [file jitc-2021-002707supp001.pdf]

Supplemental legends

Supplementary figure 1. Surface plasmon resonance (SPR) analysis of various attachment moieties used in coating of BCG. Various CPP sequences as well as cholesterol moiety were tested by surface plasmon resonance (SPR) for their efficacy at anchoring therapeutic peptides into the mycobacterial cell wall. Cady sequence GLWRALWRLLRSLWRLLWRA, Penetratin sequence RQIKIWFQNRRMKWKK, KLAL sequence KLALKLALKALKAALKLA, N-terminal cholesterol moiety and CPP Tat sequence GRKKRRQRRRPQ were compared for binding efficacy.

Supplementary figure 2. Coating BCG with CPP-containing peptide antigen but not with poly-lysine-containing peptide antigen decrease BCG viability. BCG was coated with either CPP-containing peptide antigen (CPP-OVA) or poly-lysine-containing antigen (polyK-OVA) and complexes were directly plated for colony formation. RAW-Blue cells (100.000 cells/well) were stimulated with BCG or PeptiBAC-OVA (using PolyK-OVA peptide) and the NF-kB/AP-1 activation was measured 24 hours post-infection.

Supplementary figure 3. Macrophages can cross-present antigens delivered by the PeptiBAC platform and can be polarized towards M1-like phenotype. A) Mouse bone-marrow derived macrophages were pulsed with PeptiBAC-OVA, BCG, poly-lysine-containing SIINFEKL peptide alone or left un-pulsed (Mock). Cross-presentation was determined by flow cytometry using APC-conjugated anti-H-2Kb bound to SIINFEKL. B) M2 macrophages were treated 24h with BCG, PeptiBAC-OVA, LPS (10ug/ml) or left untreated (Mock). MHC-II, CD86 and CD208 expression was determined by flow cytometry. Each bar is the mean ± SEM of technical triplicates. Statistical analysis was performed with one-way ANOVA. **** $p< 0.0001$ *** $p<0.001$.

Supplementary figure 4. Average tumour growth curves for each treatment group in B16.F10.9/K1 melanoma experiment. Statistical analysis was performed with two-way ANOVA. ** $p< 0.01$.

Supplementary figure 5. Average tumour growth curves for each treatment group in CT26 colon carcinoma experiment. Statistical analysis was performed with two-way ANOVA. * $p< 0.05$, ** $p< 0.01$.

Supplementary figure 6. Optical modelling of the maximum SPR angular response. The black curve in the figure represents the simulated SPR spectra for a native SPR sensor without BCG and the red curve depicts the simulated SPR spectra with an even homogeneous thickness of a fully covered hexagonally packed layer of BCG.

Supplementary figure 7. Histograms and gating strategies used in the analysis of flow cytometry data. A) Gating strategy used for the tumour sample analysis shown figures 5 and 6. B) Gating strategy used for the splenocyte analysis shown in figures 5 and 6. C) Gating strategy for macrophage presentation analysis shown in supplementary figure 3.
