# Supplementary figures and images for: Novel personalized cancer vaccine platform based on Bacillus Calmette-Guèrin
Source: J Immunother Cancer. 2021 Jul 15;9(7):e002707. doi: 10.1136/jitc-2021-002707 (PMC8286790; doi:10.1136/jitc-2021-002707)

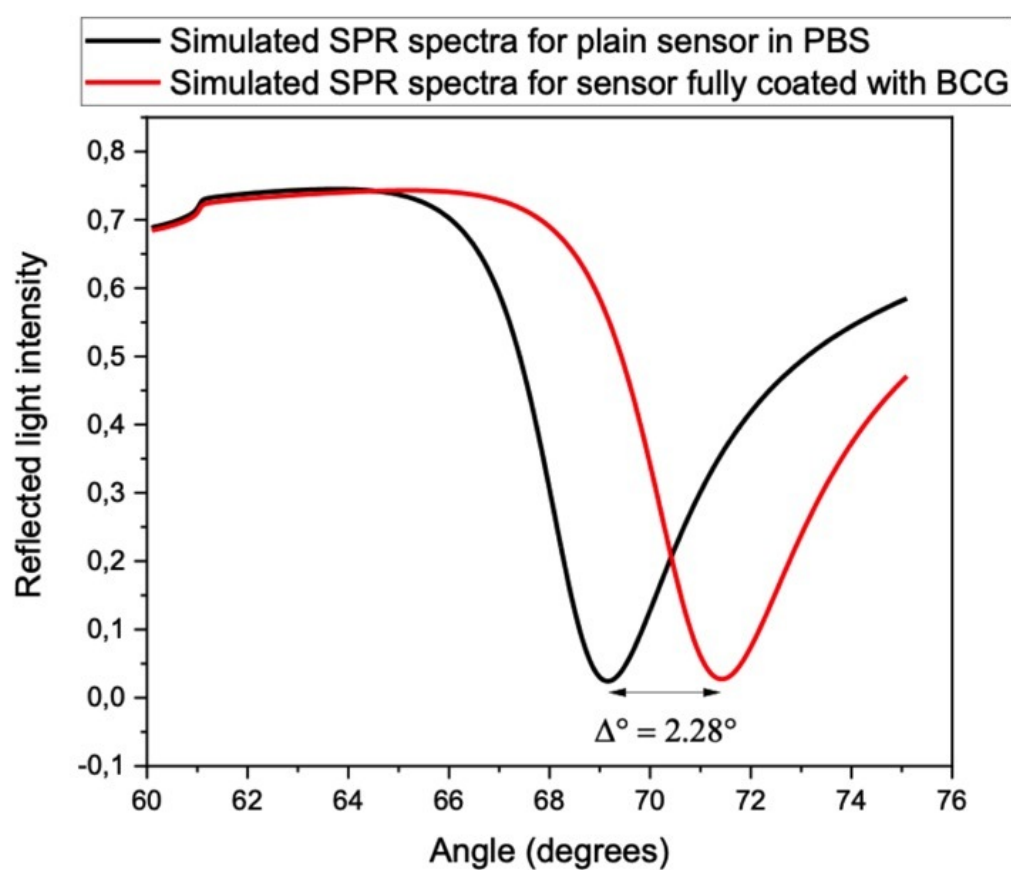

Supplement: Supplementary data [file jitc-2021-002707supp008.pdf]

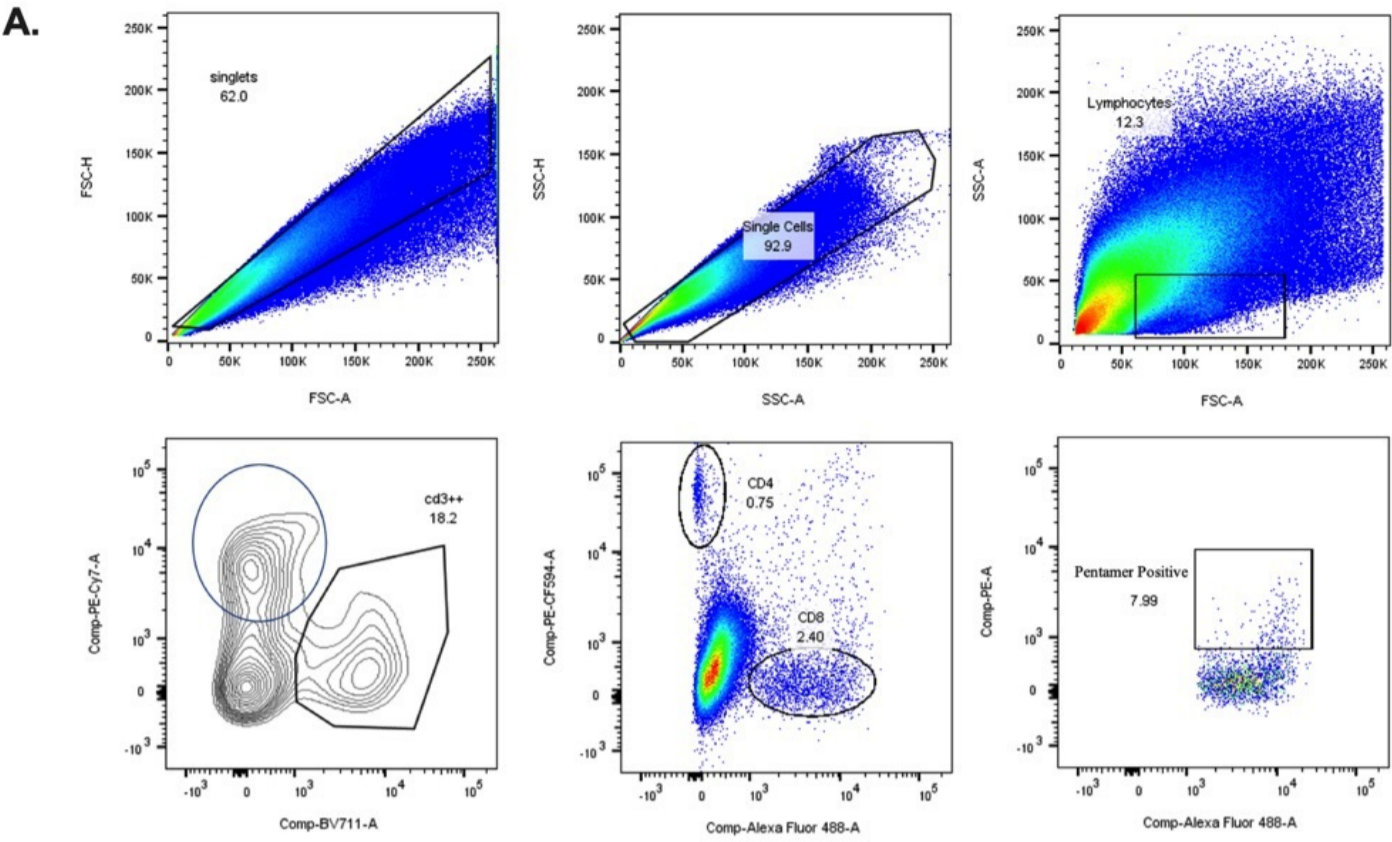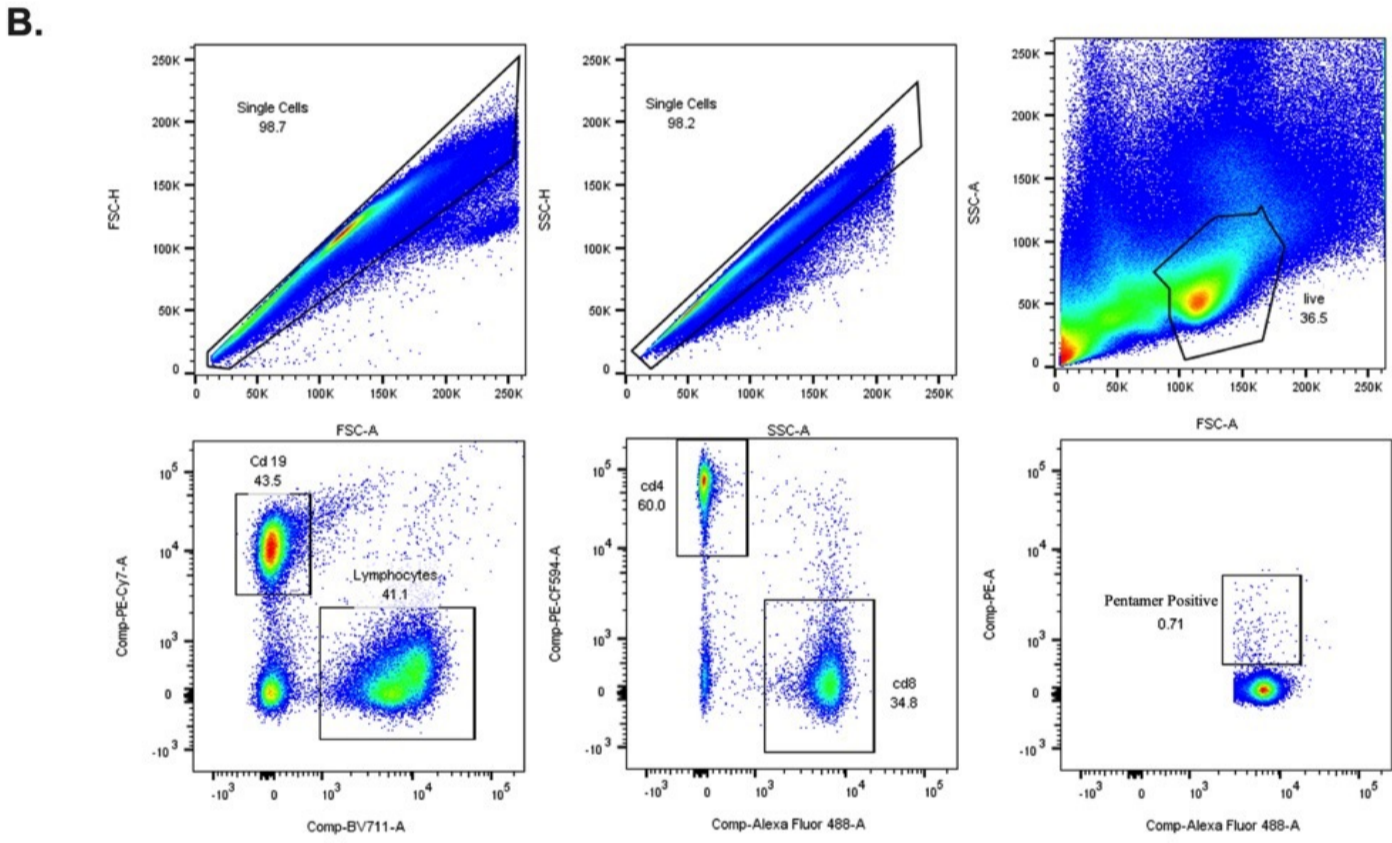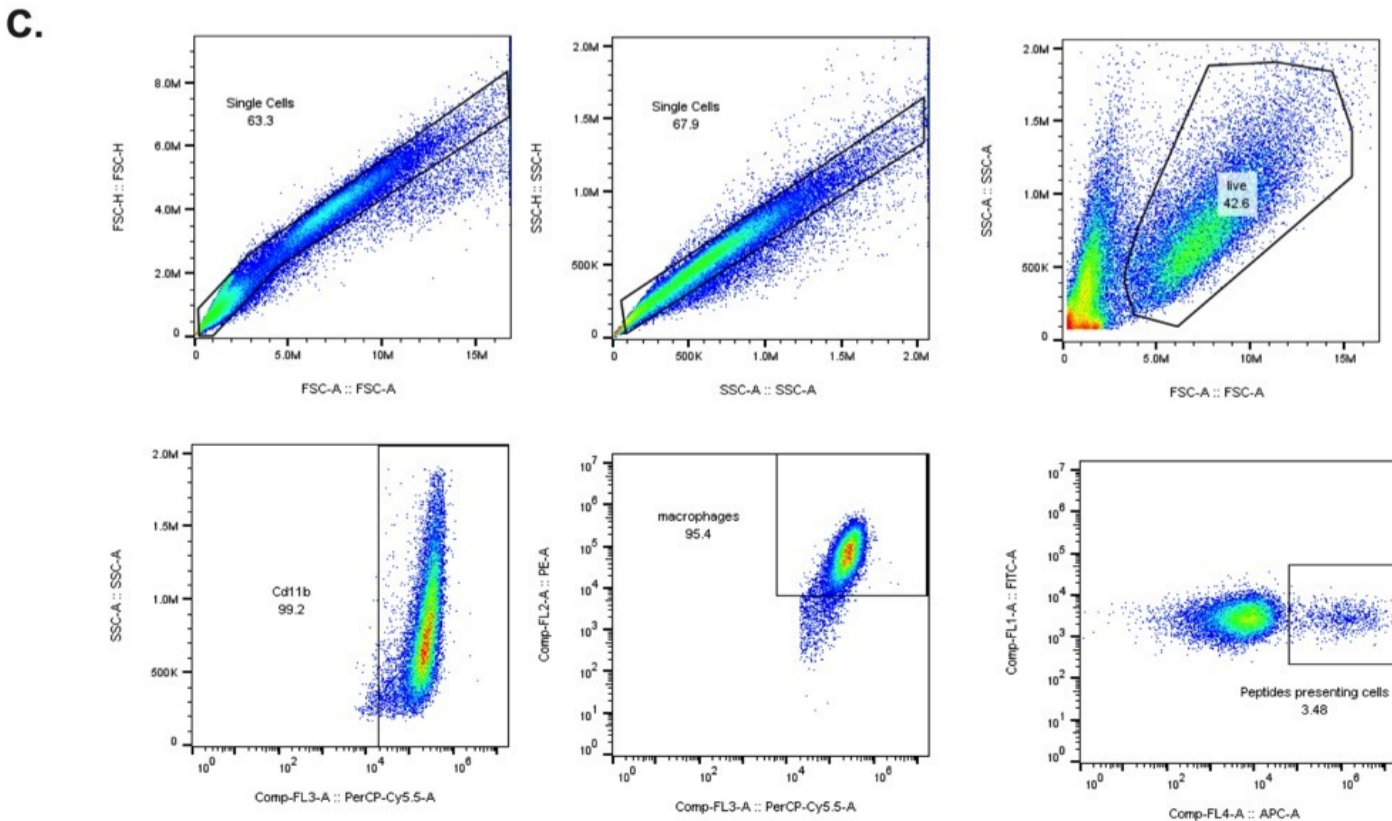

Supplement: Supplementary data [file jitc-2021-002707supp002.pdf]

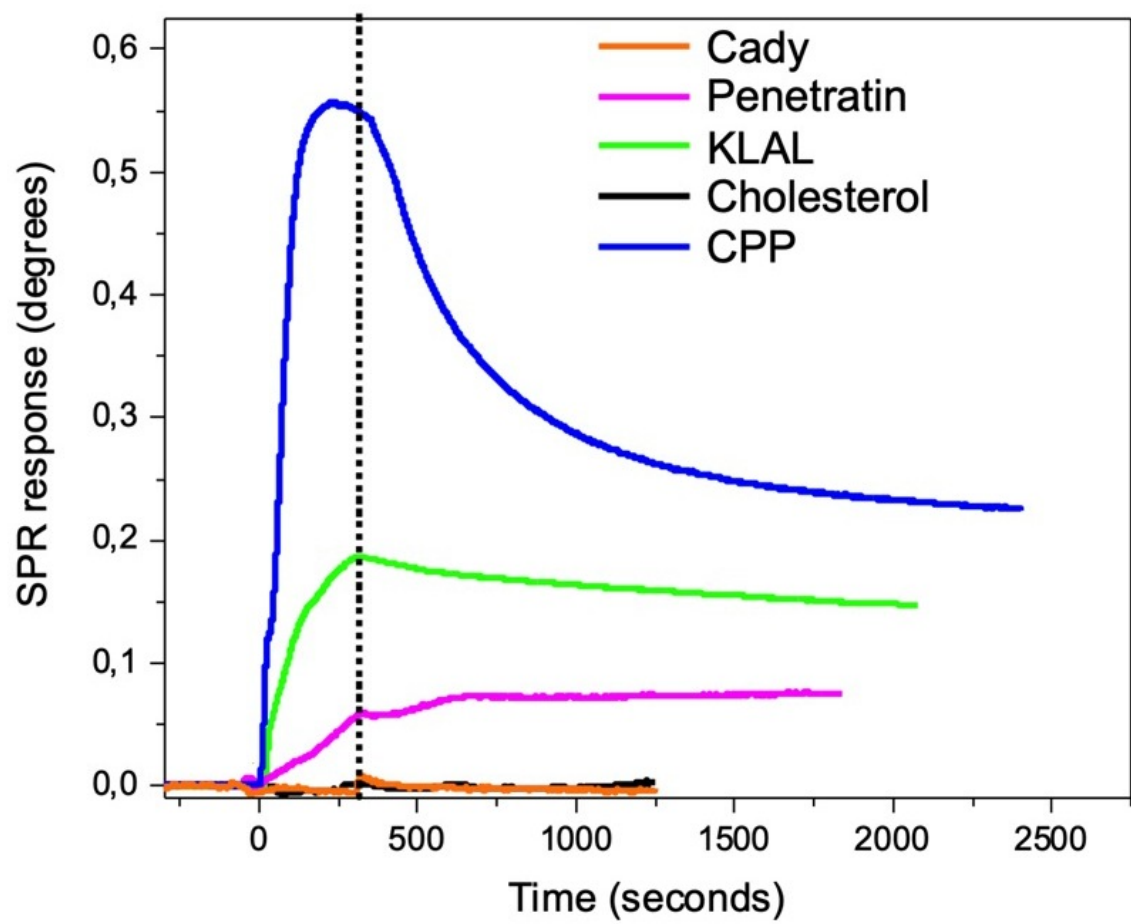

Supplement: Supplementary data [file jitc-2021-002707supp003.pdf]

Effects of peptide coating on BCG

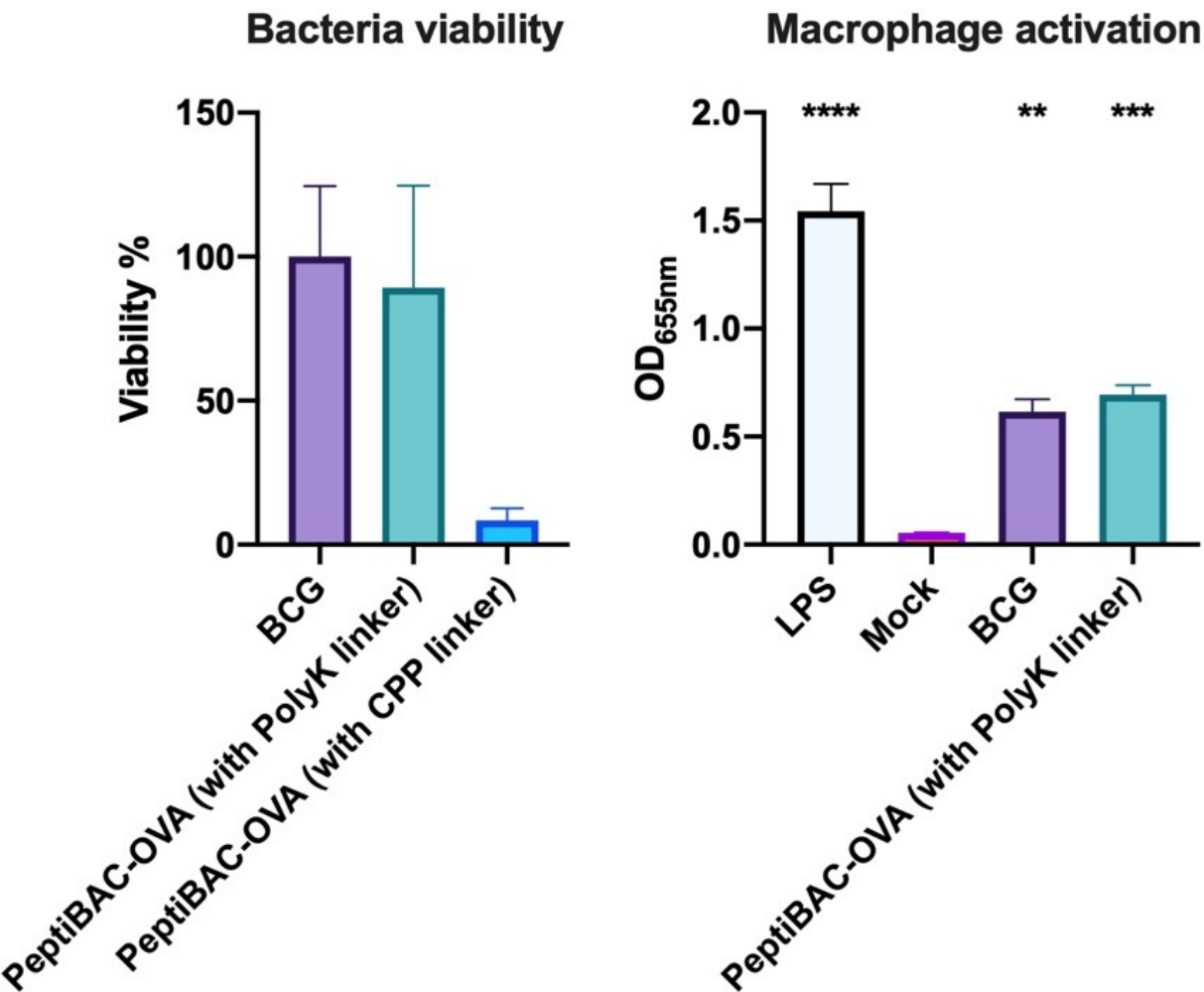

Supplement: Supplementary data [file jitc-2021-002707supp004.pdf]

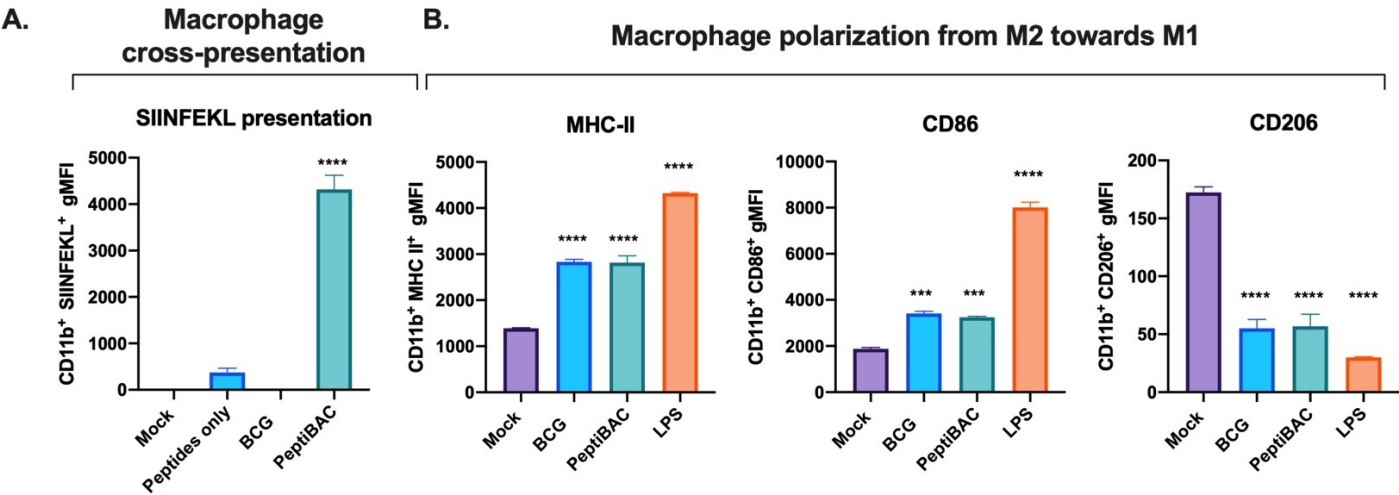

Supplement: Supplementary data [file jitc-2021-002707supp005.pdf]

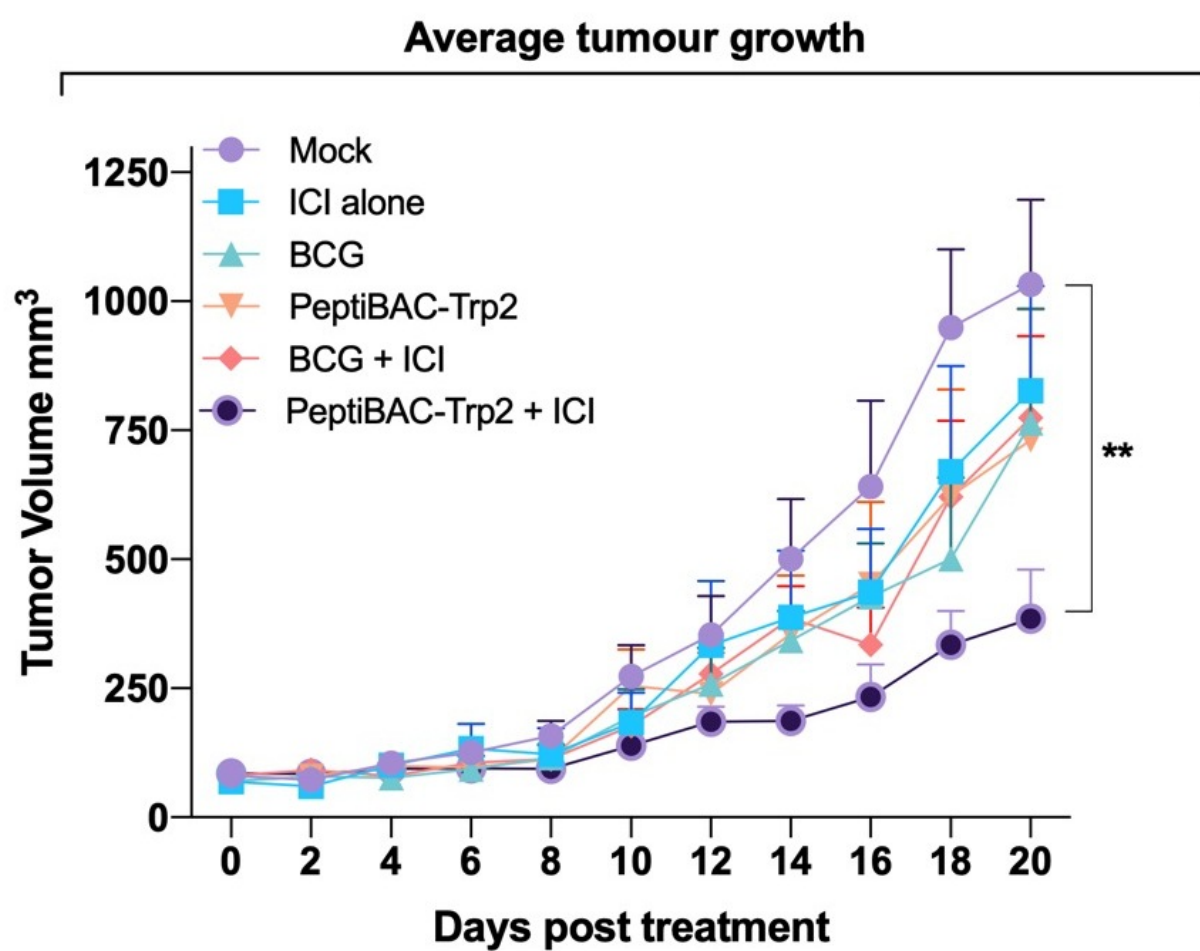

Supplement: Supplementary data [file jitc-2021-002707supp006.pdf]

## Average tumour growth

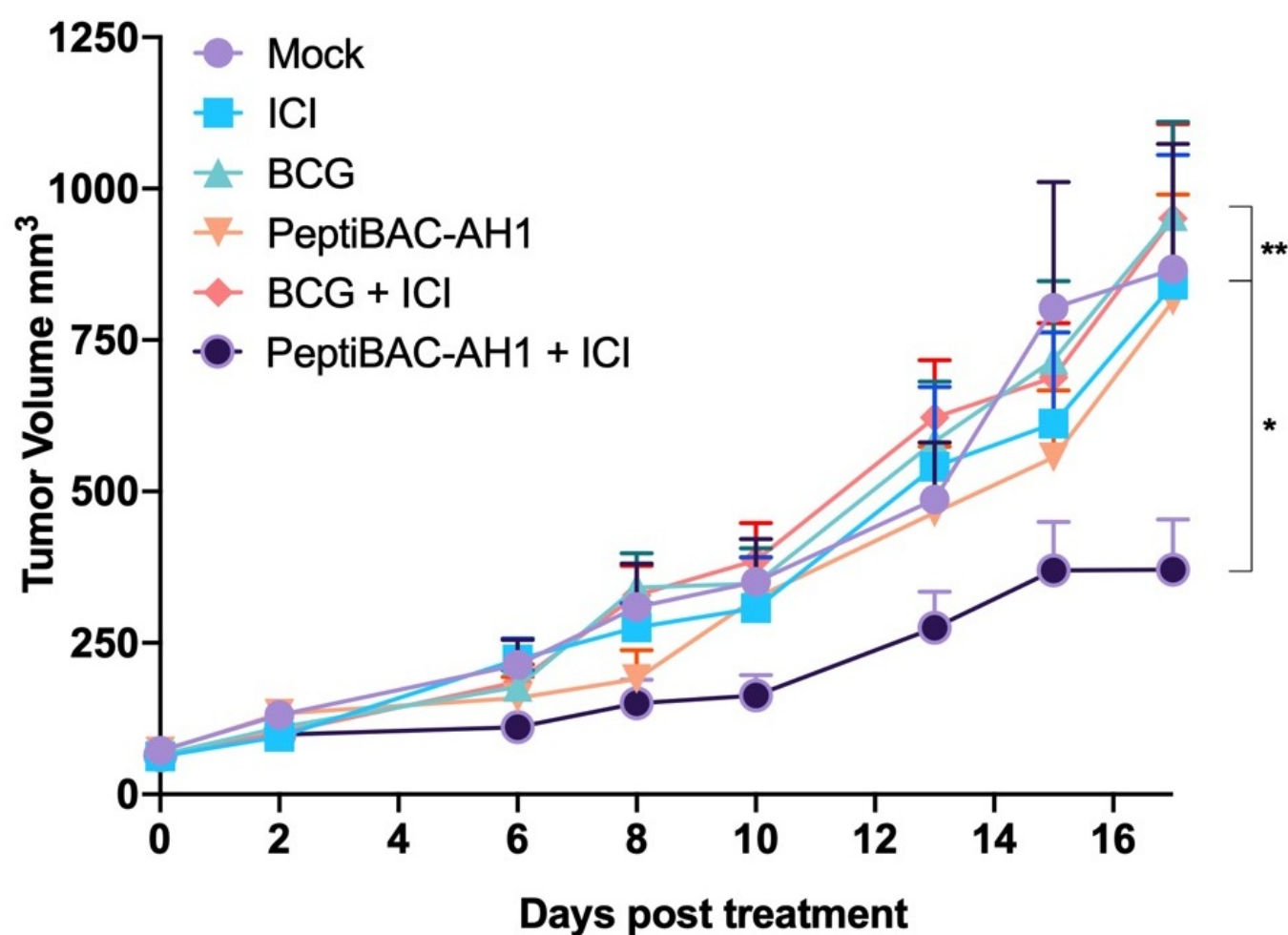

Supplement: Supplementary data [file jitc-2021-002707supp007.pdf]
